# Phosphorus Pentachloride Promoted *gem*-Dichlorination of 2′- and 3′-Deoxynucleosides

**DOI:** 10.3390/molecules23061457

**Published:** 2018-06-15

**Authors:** Fabio da Paixao Soares, Elisabetta Groaz, Piet Herdewijn

**Affiliations:** Medicinal Chemistry, Rega Institute for Medical Research, KU Leuven, Herestraat 49, 3000 Leuven, Belgium; fabio.dapaixaosoares@student.kuleuven.be (F.d.P.S.); elisabetta.groaz@kuleuven.be (E.G.)

**Keywords:** *gem*-dichlorination, modified nucleosides, halogenated nucleosides, phosphorus pentachloride

## Abstract

Halogen substitution at various positions of canonical nucleosides has generated a number of bioactive structural variants. Herein, the synthesis of two unique series of sugar modified nucleosides bearing a *gem*-dichloro group is presented. The synthetic plan entails the controlled addition of phosphorus pentachloride to suitably protected 2′- or 3′-ketodeoxynucleoside intermediates as the key step, facilitating the rapid construction of such functionalized molecules. Under the same reaction conditions, the highest chemoselectivity was observed for the formation of 2′,2′-dichloro-2′,3′-dideoxynucleosides, while a competing 2′,3′-elimination process occurred in the case of the 3′,3′-dichloro counterparts.

## 1. Introduction

**Figure 1:**
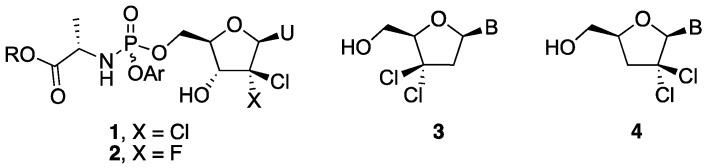
Selected biologically active (**1**–**2**) and targeted (**3**–**4**) sugar chlorinated nucleos(t)ide analogues.

One promising way to impart biologically favorable properties to natural nucleosides consists in the introduction of one or more halogen substituents into their sugar ring moiety [1,2]. In particular, the replacement of an H atom or OH group by a more electronegative chlorine atom can affect inter and/or intramolecular forces, allowing for instance additional dipolar interactions [3]. This, in combination with the potential conformational changes induced in the parent molecule by the increased atomic size of chlorine [4], may influence the binding of ligands, thus modulating the inhibition of specific targets [5]. Furthermore, the high polarizability associated with chlorine can give rise to London dispersion and consequently lipophilic properties, which may increase the passive diffusion of chlorinated nucleoside derivatives across the cell membrane. In general, chlorine has been widely employed as a bioisostere in drug development to provide a large number of therapeutic agents with a remarkable safety profile for the treatment of a variety of diseases [6]. However, to date the investigation of such structural variation in the field of nucleosides remains underexplored. Recently, phosphoramidate prodrugs of β-d-2′-deoxy-2′,2′-dichlorouridine, which were obtained upon 2′-*C*-modification of uridine with a *gem*-dichloro (CCl_2_) functionality, have been shown to have inhibitory activity against hepatitis C virus (HCV) replication (Figure 1, **1**) [7]. Additionally, Zhou et al. reported that the substitution of the 2′-methyl group in sofosbuvir with a chlorine atom generated 2′-chloro-2′-fluoro ribonucleotide prodrugs with pan-genotypic anti-HCV activity (Figure 1, **2**) [8].

Various protocols have been reported for the preparation of monochlorinated sugar modified nucleosides. Chlorine has been introduced starting from anhydro nucleosides by using either HCl-Py or HCl-dioxane in the synthesis of seven-membered ring nucleoside analogues [9] as well as 3′- and 2′-chlorinated thymidine derivatives [10,11], respectively. Alternatively, milder conditions have been employed including either a mixture of SOCl_2_ and hexamethylphosphoramide to achieve the regioselective chlorination at the 5′-position of ribonucleosides [12,13] or lithium chloride via nucleophilic displacement of sulfonyl ester derivatives as in the synthesis of 4′-chloromethyl-2′-deoxy-3′,5′-di-*O*-isobutyryl-2′-fluorocytidine, a respiratory syncytial virus (RSV) polymerase inhibitor [14,15]. Other reagents that have been successfully used are tris(2,4,6-tribromophenoxy) dichlorophosphorane (BDCP) [16] and CCl_4_/PPh_3_ [17]. However, most of these methods require multistep protecting group strategies that make them time-consuming, expensive, and low yielding. On the other hand, few efforts have been devoted to the preparation of halogenated nucleosides by condensing the base moiety with a chlorinated carbohydrate. Watanabe et al. described the synthesis of several 2′-halo-5-substituted-arabinofuranosyl derivatives upon coupling of trimethylsilylated pyrimidines with suitably protected 2-fluoro-, 2-chloro-, or 2-bromoarabinosyl bromides in good yields [18]. The glycosylation of 2-deoxy-2,2-dichlorofuranose 1-chloride with *N*^4^-Bz-cytosine has also been reported towards the synthesis of β-d-2′-deoxy-2′,2′-dichlorouridine [7]. β-2′-Chloro-α-2′-fluororibonucleoside derivatives were prepared from a β-2-chloro-α-2-fluororibofuranose intermediate, which was in turn obtained from 2-deoxy-d-ribose in five steps using *N*-chlorosuccinimide (NCS) in the presence of lithium bis(trimethylsilyl)amide (LiHMDS) in the chlorination step [8].

Herein, we describe the unprecedented synthesis of 3′,3′-*gem*-dichloro- (Figure 1, **3**) and 2′,2′-*gem*-dichloro-2′,3′-dideoxynucleoside analogues (Figure 1, **4**) bearing both purines and pyrimidines as nucleobases by using PCl_5_ as a powerful chlorinating agent. In addition, all synthesized compounds were further evaluated for their antiviral activity against human immunodeficiency virus (HIV) type 1 (III_B_ strain) and type 2 (ROD) in MT-4 cell cultures.

## 2. Results and Discussion

**Scheme 1:**
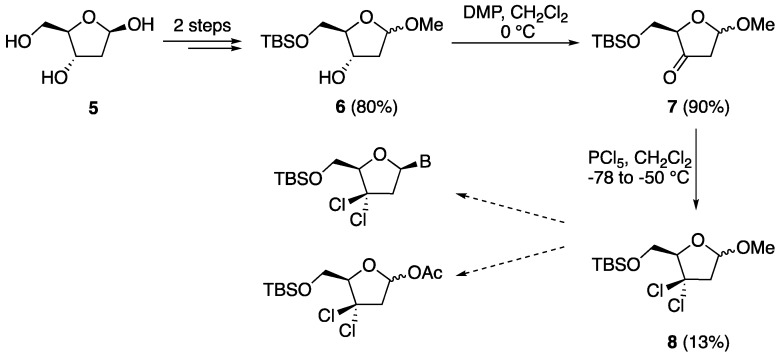
Initial attempted route towards the preparation of *gem*-dichlorinated nucleosides.

At the start of our synthetic endeavor towards the preparation of compounds of type **3** (Figure 1), we initially considered the preparation of suitably protected 3,3-*gem*-dichloro-2′,3′-dideoxyribose **8** as common synthon for further base condensation reactions (Scheme 1). The known 5-silylated 1-methoxy-2-deoxyribofuranose **6**, prepared in two-steps from 2-deoxy-d-ribose **5** [19,20,21], was easily oxidized using Dess-Martin periodinane (DMP) to provide protected 3-ketoribofuranose **7** as a stable compound in excellent yield. However, when **7** was reacted under a range of standard chlorination conditions including PCl_5_/PCl_3_ [22], Appel reaction [23,24], and the *N*-chlorosuccinimide/chlorodiphenylphosphine system [25], *gem*-dichloro sugar **8** could not be formed. Interestingly, the desired product could be obtained when the reaction was conducted in the presence of PCl_5_ at low temperature (−78 °C). Compound **8** was purified by column chromatography using neutralized silica gel, nonetheless it underwent extensive degradation and could only be isolated in a disappointing 13% yield. This is presumably due to its proclivity towards aromatization following elimination of a molecule of methanol and HCl under acid or basic conditions. In addition, the chlorinated sugar could not be converted to its acetylated derivative, while suffering further decomposition under Vorbrüggen glycosylation conditions.

**Scheme 2:**
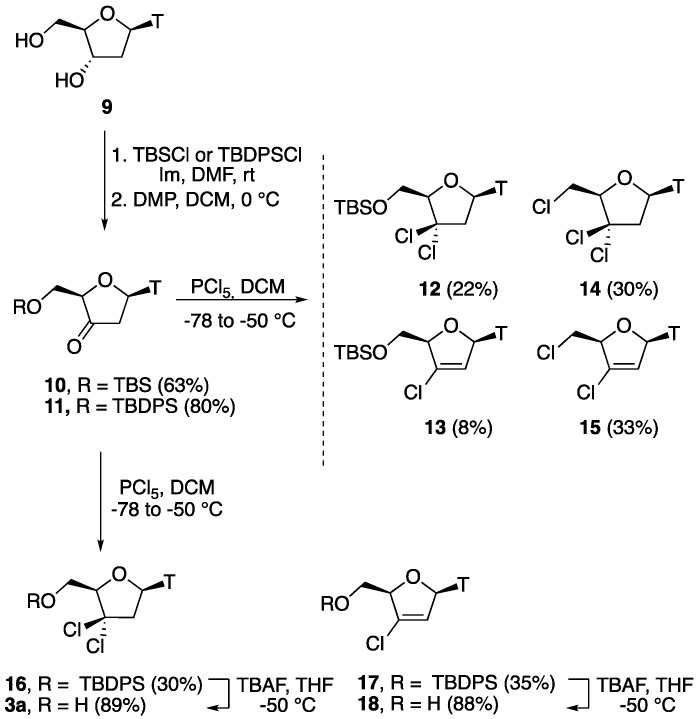
Synthesis of 3′,3′-*gem*-dicloro and 2′,3′-vinyl-3′-chloro 2′-deoxythymidine analogues.

Due to the chemical instability of 3,3-dichloro-2,3-dideoxyribofuranose **8**, this route was thus discontinued in favor of an alternative synthetic approach directly starting from 2′-deoxy-ribonucleosides. As shown in Scheme 2, 2′-deoxythymidine **9** was regioselectively silylated at the 5′-position using *tert*-butyldimethylsilyl (TBSCl) and the remaining hydroxyl functionality was then oxidized to give compound **10** in good yield. In agreement with the aforementioned results, when a variety of chlorinating conditions were screened, only the use of PCl_5_ at low temperature (−78 °C) enabled the formation of 3′,3′-*gem*-dichloro thymidine derivative **12**. However, while the *gem*-dichlorination was found to be possible, it was observed that under the employed conditions only a modest yield (22%) of the desired compound was obtained. The starting 3′-ketonucleoside **10** was in fact mainly converted to compounds **14** and **15** resulting from perchlorination reactions, which occurred after in situ deprotection of the 5′-TBS group. A small amount (8%) of an additional side product, i.e., 3′-chloro-vinyl derivative **13**, was also isolated owing to the 2′,3′-elimination of HCl from **12**. In order to improve the observed chemoselectivity, it was reasoned that the replacement of the protecting group at the 5′-position with a relatively less reactive moiety could suppress the perchlorination pathways. 5′-TBDPS-3′-ketonucleoside **11** was therefore synthesized, which led to an improved yield of the corresponding thymidine *gem*-dichloride **16** (30%) when subjected to the chlorination conditions. Although an exclusive 3′-dichlorination was ultimately not realized, compound **16** could be easily separated by column chromatography from the monochlorinated derivative **17** (ratio **16**:**17** was approximately 1:1). Both compounds were readily deprotected with tetra-*n*-butylammonium fluoride (TBAF) at −50 °C to afford the corresponding nucleosides **3a** and **18** in good yields. TBAF was added at low temperature in order to avoid fast decomposition of the starting material, which was observed when the addition was performed either at room temperature or 0 °C.

**Scheme 3:**
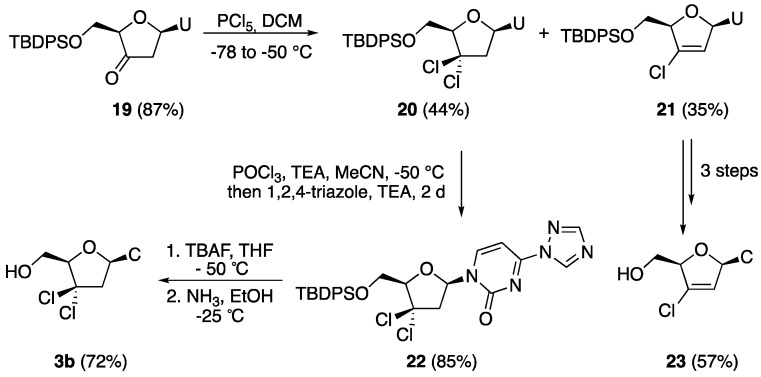
Synthesis of 3′,3′-*gem*-dicloro and 2′,3′-vinyl-3′-chloro 2′-deoxycytidine analogues.

In a similar fashion, 3′-keto-2′-deoxyribonucleoside derivative **19** bearing uracil as nucleobase was synthetized in two steps from 2′-deoxyuridine (see Materials and Methods). After the chlorination step, a 1.2:1 mixture of di- (**20**) and monochlorinated (**21**) derivatives was formed, which were isolated by subsequent chromatrographic purification and additionally submitted to pyrimidine base interconversion under standard conditions (Scheme 3). However, when the transformation into of the corresponding triazolyl intermediates (for example compound **22**) was conducted at room temperature, only poor product yields were observed due to the formation of side products originating from the nucleobase elimination. Pleasingly, it was found that by premixing **20** (and analogously **21**) with POCl_3_ in dry acetonitrile at low temperature (−50 °C) prior to the slow addition of triethylamine, followed by an acetonitrile solution containing 1,2,4-triazole and triethylamine, no degradation was observed. After desilylation and substitution at the 4-position, *gem*-dichloro compound **3b** was obtained in good yield (72%) over two steps, while compound **23** was also synthesized in 3 steps from **21** with an overall yield of 54%.

**Scheme 4:**
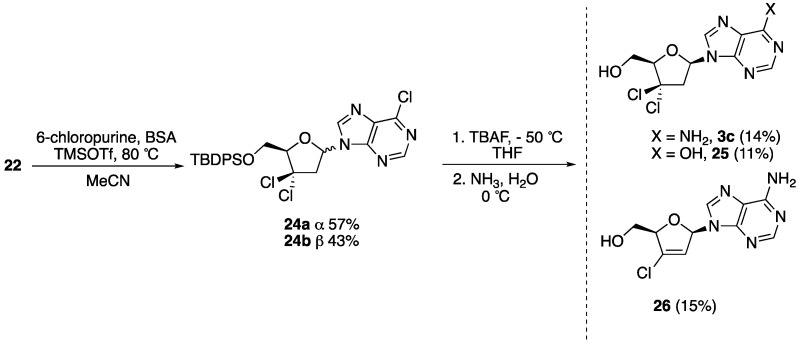
Synthesis of 3′,3′-*gem*-dichloro 2′-deoxyadenosine analogue **3c**.

Next, the previously synthesized triazolyl derivative **22** was subjected to a transglycosylation reaction [26], as depicted in Scheme 4. Thus, compound **22** was reacted with silylated 6-chloropurine in the presence of trimethylsilyl trifluoromethanosulfonate as catalyst to afford nucleoside **24** as an anomeric mixture, which could be separated by column chromatography to afford 57% and 43% of the α and β anomer, respectively. The β anomer was then reacted with TBAF to accomplish the removal of the TBDPS group, followed by displacement of the chlorine atom with NH_3_ in MeOH. The desired 3′,3′-*gem*-dichloro-2′,3′-dideoxyadenosine derivative **3c** was obtained in poor yield (14%), together with side products **25** and **26**.

**Scheme 5:**
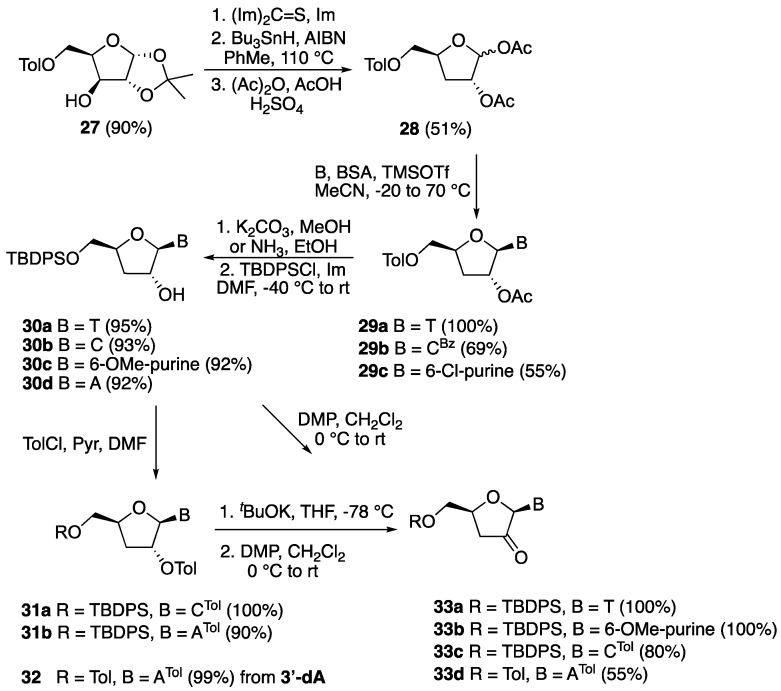
Synthesis of 2′-keto-3′-deoxynucleoside precursors.

In order to access the corresponding structural nucleoside analogues bearing a *gem*-dichloro moiety at the 2′-position (**4**, Figure 1), we envisioned an analogous sequence of reactions. In this case, the synthesis plan involved the initial preparation of a suitably protected 3-deoxyribofuranose synthon (Scheme 5). Thus, 1,2-*O*-isopropylidene-d-xylofuranose was obtained from d-(+)-xylose over two steps in one pot and multigram scale according to a previously reported procedure [27], and further protected at the 5-position to afford 5-*O*-*o*-toluoyl-1,2-isopropylidene-d-xylofuranose **27**. Subsequently, this compound was transformed into its 3-thiocarbonylimidazole derivative and subjected to Barton-McCombie deoxygenation conditions to give a 3-deoxyribofuranose intermediate, which was diacetylated using a mixture of acetic anhydride and acetic acid in the presence of a catalytic amount of sulfuric acid to furnish glycosyl donor **28** in 51% yield over 3 steps. Alternatively, TFA could also be used as catalyst to provide acetylated 3′-deoxyribofuranose **28** in higher yield (90%), but a longer reaction time was required. The *ortho*-toluoyl protecting group at the 5-position was preferred over the previously employed TBDPS functionality after that initial attempts to introduce the thiocarbonyldimidazole at the sterically hindered 3-position in the presence of such silyl group were unsuccessful.

3-Deoxyribofuranose **28** was reacted with thymine, *N*^4^-benzoylcytosine, and 6-chloropurine under Vorbrüggen glycosylation conditions to provide the corresponding 5′-*O*-*o*-toluoyl-2′-*O*-acetyl-3′-deoxynucleosides **29a**–**c**. Upon coupling with thymine, the corresponding β-anomeric nucleoside analogue **29a** was exclusively formed in quantitative yield. However, in the case of *N*^4^-benzoyl-cytosine and 6-chloropurine, 10% of the α-anomer (α:β ratio of 1:9) and 7% of the *N*^7^-alkylated regioisomer were also formed, together with other side products that were not isolated (˂5%), respectively, leading to relatively lower yields of compounds **29b** and **29c**. The assignment of the β-glycosidic bond of the 3′-deoxynucleoside analogues was carried out by 2D-NOESY experiments. The presence of a significant NOE effect between H-4′ and H-1′, and the absence of any NOE interaction between H-1′ and H-5′ at the level of the sugar moiety corroborated the stereoselectivity of the reaction for purine and pyrimidine nucleobases. In addition, the coupling constants for the anomeric protons of the synthesized 3′-deoxynucleosides were found to be in agreement with previous reports [28,29]. After standard protecting group manipulation, compounds **29a** (B = T) and **29b** (B = C^Bz^) were converted to 5′-TBDPS-3′-deoxynucleosides **30a** and **30b**. 6-Chloropurine 3′-deoxynucleoside **29c** could either undergo displacement of the chlorine atom by a methoxide functionality or be transformed into the corresponding adenine containing derivative, depending on the reaction conditions, to afford after further silylation compounds **30c** and **30d**, respectively. Silylated 3′-deoxynucleoside derivatives **30a** (B = T) and **30c** (B = 6-OMe-purine) were then oxidized using DMP in dry DCM at 0 °C to give the desired 5′-*O*-TBDPS-2′-ketonucleosides **33a** and **33b** in quantitative yield, which could be easily purified by column chromatography.

In order to obtain the corresponding cytosine- and adenine-containing nucleoside derivatives, compounds **30b** (B = C) and **30d** (B = A) were fully protected at the 2′-OH and exocyclic amino group using *o*-toluoyl chloride in the presence of pyridine. The resulting compounds **31a** and **31b** were then reacted with one equivalent of potassium *tert*-butoxide at −78 °C in dry THF. In the case of the cytosine derivative, regioselective deprotection at the 2′-position led to the desired nucleoside intermediate, which was readily oxidized to the corresponding ketone **33c**. In contrast, under similar conditions, adenine derivative **31b** underwent complete detoluylation reverting to 5′-*O*-TBDPS-3′-deoxyadenosine **30d**. However, we found that the desired transformation could be efficiently achieved after replacement of the 5′-TBDPS with a 5′-*o*-Tol group. Thus, compound **29c** was transformed into 3′-deoxyadenosine (**3′-dA**), toluoylated to **32**, which subsequently could be selectively deprotected at the 2′-position and further oxidized to give the desired 5′-*O*-*o*-toluolyl-2′-keto-6-*N*-*o*-toluoyl adenine containing nucleoside **33d** in 55% yield over two steps.

**Scheme 6:**
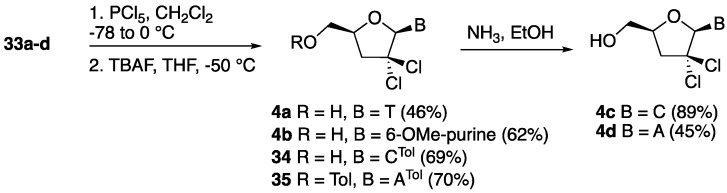
Synthesis of 2′,2′-*gem*-dichloro-2′,3′-dideoxynucleosides.

Subsequently, all suitably protected 2′-keto nucleosides **33a**–**d** were subjected to the chlorination reaction in the presence of PCl_5_ to furnish thymine and 6-OMe-purine containing 2′,2′-*gem*-dichloro nucleosides **4a** and **4b** as well as protected nucleosides **34** and **35**, which upon final removal of the toluoyl moieties with ethanolic ammonia provided the desired 2′,2′-dichloro-2′,3′-dideoxycytidine and adenosine derivatives **4c** and **4d** (Scheme 6). Although this transformation could also suffer from a concomitant elimination reaction, we did not notice any side product formation with the exception of the thymine derivative. In this case, 5′-*O*-TBDPS-2′-chloro-2′,3′-vinyl-2′,3′-dideoxythymidine derivative (19%) was readily isolated from the reaction mixture after column chromatography and characterized.

The confirmation of the structure of all intermediates and final compounds was carried out by 1D and 2D NMR spectroscopic analysis. Furthermore, mass spectroscopy in positive mode further proved the structure of the chlorinated nucleosides. The presence of chlorine atoms in the target compounds was confirmed by the intensity ratios of the isotopes of the protonated molecular ions (see Supplementary Materials). In the case of the dichloride derivatives, the molecular ions consisted of three peaks spaced by two mass units, while for monochlorinated nucleoside derivatives two peaks were observed. AQS for biological activity, unfortunately, none of the synthesized chlorinated nucleoside analogues showed any activity against HIV-1 and HIV-2 in MT-4 cell cultures.

## 3. Materials and Methods

### 3.1. General Information

All reagents and solvents were purchased from commercial sources and used as obtained. Moisture sensitive reactions were carried out in oven-dried glassware under a nitrogen or argon atmosphere unless otherwise stated. ^1^H- and ^13^C-NMR spectra were recorded on an Avance 300, 500, or 600 MHz spectrometer (Bruker BioSpin, Billerica, MA, USA) using tetramethylsilane as internal standard or referenced to the residual solvent signal. Chemical shifts (δ) are expressed in parts per million (ppm), while coupling constants in Hz (Hertz). High-resolution mass spectra (HRMS) were acquired on a quadruple orthogonal acceleration time-of-flight mass spectrometer (Synapt G2 HDMS, Waters, Milford, MA, USA). Samples were infused at 3 μL/min, and spectra were obtained in positive (or in negative) ionization mode with a resolution of 15,000 (FWHM) using leucine enkephalin as lock mass. Thin layer chromatography (TLC) was performed on silica gel Alugram (aluminum foil) pre-coated sheets (254 nm, Macherey-Nagel, Düren, Germany). Products were purified by column chromatography on silica gel (60 Å, 0.035−0.070 mm, Acros Organics, Geel, Belgium). Preparative RP-HPLC purification was performed on a Gemini 110A column (C18, 10 μm, 21.2 mm × 250 mm, Phenomenex, Utrecht, Belgium) using H_2_O/CH_3_CN as eluent gradient. Purities of all the tested compounds were verified to be >95% by HPLC analysis.

### 3.2. General Oxidation Procedure

A stirred solution of 2′- or 3′-deoxynucleoside (1.0 equiv.) in CH_2_Cl_2_ (15 mL/mmol) was cooled in an ice bath, and then Dess-Martin periodinane (DMP) (1.0 equiv.) was added under an inert atmosphere. The reaction mixture was allowed to warm to room temperature and stirred for 4 h. It was then diluted with EtOAc (50 mL/mmol) and washed successively with saturated aq. NaHCO_3_, water, and brine. The organic layer was dried over Na_2_SO_4_, filtered, and evaporated in vacuo to give a crude residue, which was purified by silica gel column chromatography to afford the title compound.

### 3.3. General Chlorination Procedure

To a stirred solution of 3′- or 2′-ketonucleoside (1 equiv.) in dry CH_2_Cl_2_ (20.0 mL/mmol) at −78 °C was added PCl_5_ (3.8 equiv.) under an inert atmosphere. The reaction mixture was allowed to warm to −50 °C and stirred for 4 h. It was then diluted with EtOAc (200.0 mL/mmol) and quenched with a K_2_HPO_4_/KH_2_PO_4_ buffer (pH = 7, 200.0 mL/mmol). Subsequently, the organic layer was separated and washed with cold water and brine. The organic layer was dried over Na_2_SO_4_, filtered, and concentrated in vacuo to give a crude residue that was purified by column chromatography to afford the title compound.

### 3.4. General Desilylation Procedure

To a stirred solution of 5′-*O*-TBDPS chlorinated nucleoside (1 equiv.) in dry THF at −50 °C was added a 1M TBAF solution in THF (1.5 equiv.). The reaction mixture was allowed to slowly warm to room temperature over 2 h. After completion of the reaction, the mixture was diluted with EtOAc and washed with water. The organic layer was dried over Na_2_SO_4_, filtered, and concentrated in vacuo to give a crude residue that was purified by column chromatography to afford the title compound.

### 3.5. General Procedure for Sugar-Base Condensation

To a solution of nucleobase (1 equiv.) in dry CH_3_CN, was added *N*,*O*-bis(trimethylsilyl)acetamide (2.5 equiv.) and the resulting mixture was stirred for 20 min at room temperature. A solution of **28** (0.8 equiv.) in dry CH_3_CN was then added and the reaction mixture was cooled to −20 °C. Next, trimethylsilyl trifluoromethanesulfonate (TMSOTf) (1.05 equiv.) was added dropwise and the mixture was allowed to slowly warm to room temperature over 1 h, heated to 70 °C, and further stirred for 1 h. It was then diluted with EtOAc and washed with saturated aq. NaHCO_3_, water, and brine. The organic layer was dried over Na_2_SO_4_, filtered, and evaporated in vacuo to give a crude residue, which was purified by silica gel column chromatography to afford the tile compound.

*5′-O-(tert-Butyldiphenylsilyl)-3′-keto-2′-deoxythymidine* (**11**). To a solution of 2′-deoxythymidine **9** (1.15 g, 4.78 mmol) and imidazole (0.650 g, 9.56 mmol) in anhydrous DMF (50 mL) was added *tert*-butyldiphenylsilyl chloride(TBDPSCl) (1.38 g, 5.00 mmol) at −50 °C. The reaction mixture was allowed to warm to room temperature and stirred for 4 h. It was then diluted with EtOAc (200 mL) and washed with water and brine. The organic layer was dried over Na_2_SO_4_, filtered, and evaporated in vacuo to give 5′-*O*-(*tert*-butyldiphenylsilyl)-2′-deoxythymidine in quantitative yield (2.28 g). ^1^H-NMR (500 MHz, CDCl_3_): δ 8.95 (s, 1H, N*H*), 7.68–7.66 (m, 4H, Ar*H*), 7.54 (d, 1H, *J* = 1.1 Hz, H-6), 7.41–7.34 (m, 6H, Ar*H*), 6.48 (dd, 1H, *J* = 8.2, 5.7 Hz, H-1′), 4.61 (br s, 1H, O*H*), 4.27–4.26 (m, 1H, H-3′), 4.10–4.08 (m, 1H, H-4′), 4.00 (dd, 1H, *J* = 11.4, 2.2 Hz, H-5′), 3.88 (dd, 1H, *J* = 11.4, 2.2 Hz, H-5″), 2.49–2.47 (m, 1H, H-2′), 2.21–2.19 (m, 1H, H-2″), 1.58 (d, *J* = 1.1 Hz, 3H, C*H*_3_), 1.09 (s, 9H, 3 × C*H*_3_); ^13^C-NMR (125 MHz, CDCl_3_): δ 164.2 (C=O), 150.6 (C=O), 135.3 (Ar*C*), 135.2 (Ar*C*), 135.0 (6-C), 132.8 (Ar*C*), 132.1 (Ar*C*), 129.8 (Ar*C*), 129.7 (Ar*C*), 127.7 (Ar*C*), 127.6 (Ar*C*), 110.8 (5-C), 87.1 (1′-*C*H), 84.5 (4′-*C*H), 71.3 (3′-*C*H), 64.0 (5′-*C*H_2_), 40.7 (2′-*C*H_2_), 31.3 (-*C*(CH_3_)_3_), 26.7 (3 × *C*H_3_), 11.8 (5-*C*H_3_). Following the general oxidation procedure, 5′-*O*-(*tert*-butyldiphenylsilyl)-2′-deoxythymidine (1.54 g, 3.00 mmol) was reacted with DMP (1.70 g, 4.00 mmol) in CH_2_Cl_2_ (100 mL). After work-up, the resulting crude residue was then recrystallized from CHCl_3_ to provide **11** as a white solid (1.14 g, 80%, over two steps), which was used in the next step without any further purification. HRMS: C_26_H_30_N_2_O_5_ [M + Na^+^]^+^ Calc.: 501.1816, found: 501.1886.

*5′-O-(tert-Butyldiphenylsilyl)-3′*,*3′-gem-dichloro-3′-deoxythymidine* (**16**) *and 5′-O-(tert-butyldiphenylsilyl)-3′-chloro-2′*,*3′-didehydro-3′-deoxythymidine* (**17**). Following the general chlorination procedure, a solution of compound **11** (0.500 g, 1.04 mmol) in dry CH_2_Cl_2_ (20 mL) was reacted with PCl_5_ (0.822 g, 3.95 mmol) at −78 °C under an inert atmosphere. After work-up, the resulting crude residue was purified by column chromatography (hexane: EtOAc 4:1) to give **16** (0.165 g, 30%) and **17** (0.180 g, 35%) as pale yellow solids. Data for **16**: ^1^H-NMR (500 MHz, CDCl_3_): δ 10.3 (s, 1H, N*H*), 7.68–7.66 (m, 4H, Ar*H*), 7.54 (d, 1H, *J* = 1.1 Hz, H-6), 7.41–7.35 (m, 6H, Ar*H*), 6.48 (dd, 1H, *J* = 8.2, 5.7 Hz, H-1′), 4.10 (dd, 1H, *J* = 4.7, 2.2 Hz, H-4′), 3.99 (dd, 1H, *J* = 11.5, 2.2 Hz, H-5′), 3.88 (dd, 1H, *J* = 11.5, 4.7 Hz, H-5″), 3.79 (dd, 1H, *J* = 11.5, 5.7 Hz, H-2′), 3.35 (dd, 1H, *J* = 11.5, 8.2 Hz, H-2″), 1.54 (s, 3H, 5-C*H*_3_), 0.97 (s, 9H, 3 × C*H*_3_); ^13^C-NMR (150 MHz, CDCl_3_): δ 163.7 (C=O), 150.2 (C=O), 135.4 (6-C), 135.4 (Ar*C*), 135.3 (Ar*C*), 135.3 (Ar*C*), 135.1 (Ar*C*), 129.7 (Ar*C*), 129.6 (Ar*C*), 127.6 (Ar*C*), 127.5 (Ar*C*), 110.8 (5-C), 87.5 (4′-*C*H), 85.0 (3′-*C*(Cl)_2_), 84.5 (1′-*C*H), 63.7 (5′-*C*H_2_), 41.0 (2′-*C*H_2_), 26.6 (3 × *C*H_3_), 19.0 (*C*(CH_3_)), 11.6 (5-*C*H_3_); HRMS: C_26_H_30_N_2_O_4_SiCl_2_ [M + H^+^]^+^ Calc.: 533.1424, found: 533.1442. Data for **17**: ^1^H-NMR (500 MHz, CDCl_3_): δ 9.00 (s, 1H, NH), 7.67 (d, 2H, *J* = 6.7 Hz, Ar*H*), 7.62 (d, 2H, *J* = 6.7 Hz, Ar*H*), 7.43–7.25 (m, 7H, Ar*H*, H-6), 7.02 (dd, 1H, *J* = 4.0, 1.3 Hz, H-1′), 5.93 (t, 1H, *J* = 1.3 Hz, H-2′), 4.78–4.77 (m, 1H, H-4′), 4.09–4.08 (m, 2H, H-5′, H-5″), 1.17 (s, 3H, 5-C*H*_3_), 1.09 (s, 9H, 3 × C*H*_3_); ^13^C-NMR (125 MHz, CDCl_3_): δ 163.7 (C=O), 150.7 (C=O), 136.3 (6-C), (3′-*C*(Cl)), 135.3 (Ar*C*), 135.2 (Ar*C*), 133.5 (Ar*C*), 132.6 (Ar*C*), 130.1 (Ar*C*), 129.9 (Ar*C*), 127.9 (Ar*C*), 127.8 (Ar*C*), 121.2 (2′-*C*H), 111.6 (5-C), 87.5 (4′-*C*H), 86.5 (1′-*C*H), 62.1 (5′-*C*H_2_), 27.1 (3 × *C*H_3_), 19.6 (*C*(CH_3_)), 11.3 (5-*C*H_3_); HRMS: C_26_H_29_N_2_O_4_SiCl [M + Na^+^]^+^ Calc.: 519.14775, found: 519.1468.

*3′*,*3′-gem-Dichloro-3′-deoxythymidine* (**3a**). Following the general desilylation procedure, a solution of compound **16** (0.143 g, 0.27 mmol) in dry THF (2.0 mL) was reacted with TBAF (1 mL, 2 mmol) at −50 °C. After work-up, the resulting crude residue was purified by column chromatography (EtOAc) to give **3a** as a yellow solid (76 mg, 89%). ^1^H-NMR (600 MHz, DMSO-d_6_): δ 11.4 (s, 1H, N*H*), 7.70 (d, 1H, *J* = 1.2 Hz, H-6), 6.27 (dd, 1H, *J* = 7.0, 6.3 Hz, H-1′), 5.34 (t, *J* = 5.0 Hz, 1H, O*H*), 4.38 (dd, 1H, *J* = 5.1, 3.1 Hz, 4′-H), 3.82 (ddd, 1H, *J* = 12.1, 5.1, 3.1 Hz, H-5′), 3.78 (ddd, 1H, *J* = 12.1, 5.0, 3.1 Hz, H-5″), 3.21 (dd, *J* = 14.7, 6.3 Hz, 1H, H-2′), 3.03 (dd, *J* = 14.6, 7.0 Hz, 1H, H-2″), 1.78 (d, 3H, *J* = 1.2 Hz, 5-C*H*_3_); ^13^C-NMR (150 MHz, DMSO): δ 163.7 (C=O), 150.5 (C=O), 135.6 (6-C), 109.8 (5-C), 89.0 (4′-*C*H), 86.9 (3′-*C*(Cl)_2_), 81.8 (1′-*C*H), 60.7 (5′-*C*H_2_), 50.4 (2′-*C*H_2_), 12.4 (5-*C*H_3_); HRMS: C_10_H_12_Cl_2_N_2_O_4_ [M + H^+^]^+^ Calc.: 317.0066, found: 317.0074.

*3′-Chloro-2′*,*3′-didehydro-3′-deoxythymidine* (**18**). Following the general desilylation procedure, a solution of compound **17** (0.124 g, 0.25 mmol) in dry THF (2.0 mL) was reacted with TBAF (1 mL, 2 mmol) at −50 °C. After work-up, the resulting crude residue was purified by column chromatography (EtOAc) to give **18** (62.6 mg, 88%) as a yellow solid. ^1^H-NMR (600 MHz, CDCl_3_): δ 8.87 (s, 1H, N*H*), 7.72 (d, 1H, *J* = 1.4 Hz, H-6), 7.01 (dd, 1H, *J* = 4.7, 1.7 Hz, H-1′), 5.96 (t, 1H, *J* = 1.7 Hz, H-2′), 5.30 (s, 1H, O*H*), 5.00–4.99 (m, 1H, 4′-H), 3.93 (dd, 1H, *J* = 15.5, 3.0 Hz, H-5′), 3.89 (dd, 1H, *J* = 15.5, 3.0 Hz, H-5″), 1.91 (d, 3H, *J* = 1.4 Hz, C*H*_3_); ^13^C-NMR (150 MHz, CDCl_3_): δ 163.5 (C=O), 150.7 (C=O), 135.9 (3′-*C*(Cl)), 135.7 (6-C), 122.9 (2′-C), 111.9 (5-C), 87.5 (4′-*C*H), 84.1 (1′-*C*H), 44.1 (5′-*C*H_2_), 12.4 (5-*C*H_3_); HRMS: C_10_H_11_Cl_1_N_2_O_4_ [M + H^+^]^+^ Calc.: 259.0480, found: 259.0489.

*5′-O-(tert-Butyldiphenylsilyl)-3′-keto-2′-deoxyuridine* (**19**). To a solution of uridine (1.09 g, 4.78 mmol) and imidazole (0.650 g, 9.56 mmol) in anhydrous DMF (50 mL) was added TBDPSCl (1.38 g, 5.00 mmol) at −50 °C. The reaction mixture was allowed to warm to room temperature and stirred for 4 h. It was then diluted with EtOAc (200 mL) and washed with water and brine. The organic layer was dried over Na_2_SO_4_, filtered, and evaporated in vacuo to give 5′-*O*-(*tert*-butyldiphenylsilyl) uridine in quantitative yield (2.21 g). ^1^H-NMR (500 MHz, CDCl_3_): δ 10.1 (s, 1H, N*H*), 7.83 (d, 1H, *J* = 8.1 Hz, H-6), 7.66–7.63 (m, 4H, Ar*H*), 7.42–7.37 (m, 6H, Ar*H*), 6.37 (t, 1H, *J* = 6.4 Hz, H-1′), 5.43 (d, 1H, *J* = 8.1 Hz, H-5), 4.56 (br s, 1H, O*H*), 4.11–4.10 (m, 1H, H-3′), 4.03–4.02 (m, 1H, H-4′), 3.98 (dd, 1H, *J* = 11.4, 2.3 Hz, H-5′), 3.85 (dd, 1H, *J* = 11.4, 2.9 Hz, H-5″), 2.49–2.44 (m, 1H, H-2′), 2.24–2.18 (m, 1H, H-2″), 1.07 (s, 9H, 3 × C*H*_3_); ^13^C-NMR (125 MHz, CDCl_3_): δ 163.7 (C=O), 150.4 (C=O), 140.0 (6-C), 135.4 (Ar*C*), 135.1 (Ar*C*), 132.6 (Ar*C*), 132.1 (Ar*C*), 129.8 (Ar*C*), 129.8 (Ar*C*), 127.7 (Ar*C*), 127.7 (Ar*C*), 102.0 (5-C), 87.0 (1′-*C*H), 84.8 (4′-*C*H), 70.8 (3′-*C*H), 63.6 (5′-*C*H_2_), 41.0 (2′-*C*H_2_), 31.3 (-*C*(CH_3_)_3_), 26.7 (3 × *C*H_3_); HRMS for C_25_H_30_N_2_O_4_Si_1_ [M + Na]^+^ Calc.: 489.1816, found: 489.1816. Following the general oxidation procedure, 5′-*O*-(*tert*-butyldiphenylsilyl) uridine (1.39 g, 3.00 mmol) was reacted with DMP (1.70 g, 4.00 mmol) in CHCl_3_ (100 mL). After work-up, the resulting crude residue was recrystallized from CHCl_3_ to provide **19** as a white solid (1.23 g, 87% over two steps), which was used in the next step without any further purification. HRMS: C_25_H_28_N_2_O_5_ [M + Na^+^]^+^ Calc.: 487.1659 found: 487.1686.

*5′-O-(tert-Butyldiphenylsilyl)-3′*,*3′-gem-dichloro-2′*,*3′-dideoxyuridine* (**20**) *and 5′-O-(tert-butyldiphenyl-silyl)-3′-chloro-2′,3′-didehydro-2′,3′-dideoxyuridine* (**21**). Following the general chlorination procedure, a solution of compound **19** (0.482 g, 1.04 mmol) in dry CH_2_Cl_2_ (20 mL) was reacted with PCl_5_ (0.822 g, 3.95 mmol) at −78 °C under an inert atmosphere. After work-up, the resulting crude residue was purified by column chromatography (hexane:EtOAc 4:1) to give **20** (0.227 g, 44%) and **21** (0.175 g, 35%) as yellow pale solids. Data for **20**: ^1^H-NMR (500 MHz, CDCl_3_): δ 9.57 (s, 1H, NH), 7.70–7.66 (m, 4H, Ar*H*), 7.56 (d, 1H, *J* = 8.2 Hz, H-6), 7.47–7.38 (m, 6H, Ar*H*), 6.23 (dd, 1H, *J* = 6.8, 4.9 Hz, H-1′), 5.51 (d, 1H, *J* = 8.2 Hz, H-5), 4.43 (dd, 1H, *J* = 5.1, 3.0 Hz, H-4′), 4.15 (dd, 1H, *J* = 11.8, 3.0 Hz, H-5′), 4.03 (dd, 1H, *J* = 11.8, 5.2 Hz, H-5″), 3.23 (dd, 1H, *J* = 14.8, 6.9 Hz, H-2′), 2.96 (dd, 1H, *J* = 14.8, 4.8 Hz, H-2″), 1.08 (s, 9H, 3 × CH_3_); ^13^C-NMR (125 MHz, CDCl_3_): δ 163.2 (C=O), 150.2 (C=O), 139.4 (6-C), 135.7 (Ar*C*), 135.4 (Ar*C*), 132.7 (Ar*C*), 132.2 (Ar*C*), 130.0 (Ar*C*), 130.0 (Ar*C*), 127.9 (Ar*C*), 127.8 (Ar*C*), 102.1 (5-C), 89.9 (4′-CH), 84.5 (3′-C(Cl)_2_), 83.3 (1′-CH), 63.1 (5′-CH_2_), 52.7 (2′-CH_2_), 29.6 (-C(CH_3_)_3_), 26.8 (3 × CH_3_); HRMS: C_25_H_28_Cl_2_N_2_O_4_Si [M + H^+^]^+^ Calc.: 519.1268, found: 519.1290. Data for **21**: ^1^H-NMR (600 MHz, CDCl_3_): δ 8.44 (s, 1H, N*H*), 7.81 (d, 1H, *J* = 8.2 Hz, 6-H), 7.66–7.64 (m, 3H, Ar*H*), 7.57–7.55 (m, 2H, Ar*H*), 7.44–7.35 (m, 5H, Ar*H*), 7.03 (dd, 1H, *J* = 3.8, 1.5 Hz, H-1′), 5.9 (t, 1H, *J* = 1.5 Hz, H-2′), 4.86 (d, 1H, *J* = 8.2 Hz, 5-H), 4.80 (dddd, 1H, *J* = 3.8, 2.7, 1.5, 1.3 Hz, H-4′), 4.07 (dd, 1H, *J* = 12.3, 1.3 Hz, H-5′), 4.07 (dd, 1H, *J* = 12.3, 2.7 Hz, H-5″), 1.10 (s, 9H, 3 × C*H*_3_); ^13^C-NMR (150 MHz, CDCl_3_): δ 158.9 (C=O), 149.7 (C=O), 136.9 (6-C), 136.5 (3′-*C*(Cl)), 136.5 (Ar*C*), 135.5 (Ar*C*), 135.2 (Ar*C*), 132.9 (Ar*C*), 132.3 (Ar*C*), 130.0 (Ar*C*), 129.9 (Ar*C*), 127.8 (Ar*C*), 127.7 (Ar*C*), 121.7 (2′-*C*H), 109.9 (5-C), 88.3 (4′-*C*H), 86.9 (1′-*C*H), 62.9 (5′-*C*H_2_), 29.6 (*C*(CH_3_), 27.1 (3 × -*C*H_3_); HRMS: C_25_H_27_ClN_2_O_4_Si [M + Na^+^]^+^ Calc.: 505.1321, found: 505.1321.

*5′-O-(tert-Butyldiphenylsilyl)-3′*,*3′-dichloro-2′*,*3′-dideoxy-4-(1H-1*,*2*,*4-triazol-1-yl)uridine* (**22**). To a stirred solution of **20** (1.04 g, 2.00 mmol) in dry CH_3_CN (10 mL) was added POCl_3_ (0.28 mL, 3 mmol) at −50 °C. Triethylamine (0.7 mL, 5.00 mmol) was then added dropwise over 10 min, followed by a solution of 1,2,4-triazole (0.414 g, 6.00 mmol) and triethylamine (0.8 mL, 6.00 mmol) in dry CH_3_CN (15 mL) over 30 min. The reaction mixture was stirred at room temperature for two days. It was then diluted with EtOAc (200.0 mL) and quenched with a NaH_2_PO_4_/Na_2_HPO_4_ buffer (pH = 7, 150.0 mL). The organic layer was washed with water and brine, dried over Na_2_SO_4_, and evaporated in vacuo to give a crude residue, which was purified by column chromatography (EtOAc:hexane 7:3) to afford compound **22** as a white solid (0. 967 mg, 85%). ^1^H-NMR (300 MHz, CDCl_3_): δ 9.19 (s, 1H, N-C*H*=N), 8.06 (s, 1H, N-C*H*=N), 7.98 (d, 1H, *J* = 7.3 Hz, H-6), 7.68–7.62 (m, 4H, Ar*H*), 7.43–7.42 (m, 6H, Ar*H*), 6.81 (d, 1H, *J* = 7.3 Hz, H-5), 6.10 (dd, 1H, *J* = 7.3, 3.0 Hz, H-1′), 4.49 (dd, 1H, *J* = 6.0, 3.0 Hz, H-4′), 4.19 (dd, 1H, *J* = 11.8, 3.0 Hz, H-5′), 4.01 (dd, 1H, *J* = 11.8, 6.0 Hz, H-5″), 3.38 (dd, 1H, *J* = 15.3, 7.3 Hz, H-2′), 3.05 (dd, 1H, *J* = 15.3, 3.0 Hz, H-2″), 1.03 (s, 9H, 3 × C*H*_3_); ^13^C-NMR (75 MHz, CDCl_3_): δ 159.8 (C=O), 154.4 (4-CN), 146.6 (N-*C*H=N), 143.6 (N-*C*H=N), 136.1 (6-C), 136.0 (Ar*C*), 135.8 (Ar*C*), 132.9 (Ar*C*), 132.7 (Ar*C*), 130.4 (Ar*C*), 130.3 (Ar*C*), 128.2 (Ar*C*), 128.1 (Ar*C*), 94.4 (5-C), 90.9 (4′-*C*H), 86.4 (1′-*C*H), 84.3 (3′-*C*(Cl)_2_), 63.2 (5′-*C*H_2_), 53.3 (2′-*C*H_2_), 30.0 (*C*(CH_3_)_3_), 27.1 (3 × *C*H_3_); HRMS: C_27_H_29_Cl_2_N_5_O_3_Si [M + H^+^]^+^ Calc.: 570.1489, found: 570.1489.

*3′*,*3′-gem-Dichloro-2′*,*3′-dideoxycytidine* (**3b**). Following the general desilylation procedure, **22** (0.142 g, 0.25 mmol) was reacted with a solution of TBAF in THF (0.4 mL, 0.375 mmol) in dry THF (2.0 mL). After work-up, the resulting crude residue was purified by column chromatography (EtOAc) to give 3′,3′-*gem*-dichloro-2′,3′-dideoxy-4-(1*H*-1,2,4-triazol-1-yl)uridine as a yellow solid (66 mg, 80%). ^1^H-NMR (500 MHz, DMSO-d_6_): δ 9.44 (s, 1H, N-C*H*=N), 8.61 (d, 1H, *J* = 7.3 Hz, H-6), 8.41 (s, 1H, N-C*H*=N), 7.03 (d, 1H, *J* = 7.3 Hz, H-5), 6.15 (dd, 1H, *J* = 7.4, 3.6 Hz, H-1′), 5.35 (s, 1H, O*H*), 4.56 (dd, 1H, *J* = 5.9, 3.1 Hz, H-4′), 3.91 (dd, 1H, *J* = 12.3, 3.1 Hz, H-5′), 3.88 (dd, 1H, *J* = 12.3, 5.8 Hz, H-5″), 3.50 (dd, *J* = 14.9, 7.4 Hz, 1H, H-2′), 3.06 (dd, *J* = 14.9, 3.6 Hz, 1H, H-2″); ^13^C-NMR (125 MHz, DMSO-d_6_): δ 159.9 (2-C=O), 154.2 (4-*C*N), 153.5 (N-*C*H=N), 148.1 (N-*C*H=N), 143.8 (6-C), 140.1 (5-C), 93.8 (4′-*C*H), 90.0 (1′-*C*H), 85.9 (3′-*C*(Cl)_2_), 60.3 (5′-*C*H_2_), 51.7 (2′-*C*H_2_); HRMS: C_11_H_11_Cl_2_N_5_O_3_ [M + Na^+^]^+^ Calc.: 354.0131, found: 354.0132. To a stirred solution of the above compound (0.100 g, 0.30 mmol,) in EtOH (3 mL) at −20 °C was added a solution of NH_3_ in EtOH, and then the reaction was allowed to warm to room temperature. After stirring for 4 h, the volatiles were removed in vacuo, and the crude residue was purified by column chromatography (EtOAc:EtOH 75:25) to give **3b** as a pale green solid (0.753 g, 90%). ^1^H-NMR (600 MHz, DMSO-d_6_): δ 7.78 (d, 1H, *J* = 7.5 Hz, H-6), 7.34 (s, 1H, N*H*_2_), 7.19 (s, 1H, N*H*_2_), 6.19 (dd, 1H, *J* = 7.1, 5.2 Hz, H-1′), 5.78 (d, 1H, *J* = 7.5 Hz, H-5), 5.32 (t, 1H, *J* = 5.3 Hz, O*H*), 4.37 (dd, 1H, *J* = 5.0, 3.0 Hz, H-4′), 3.83 (ddd, 1H, *J* = 12.1, 5.3, 3.0 Hz, H-5′), 3.77 (ddd, 1H, *J* = 12.1, 5.3, 5.0 Hz, H-5″), 3.25 (dd, 1H, *J* = 14.7, 7.2 Hz, H-2′), 2.87 (dd, 1H, *J* = 14.6, 5.2 Hz, H-2″); ^13^C-NMR (150 MHz, DMSO-d_6_): δ 165.7 (4-*C*-NH_2_), 154.9 (2-C=O), 140.65 (6-C), 94.2 (5-C), 89.1 (4′-C), 86.80 (3′-*C*(Cl)_2_), 83.1 (1′-*C*H), 60.6 (5′-*C*H_2_), 51.6 (2′-*C*H_2_); HRMS: C_9_H_10_Cl_1_N_3_O_3_ [M + H^+^]^+^ Calc.: 280.0250, found: 280.0250.

*3′-Chloro-2′*,*3′-didehydro-2′*,*3′-dideoxycytidine* (**23**). To a stirred solution of **21** (0.95 g, 2.00 mmol) in dry CH_3_CN (10 mL) was added POCl_3_ (0.28 mL, 3 mmol) at −50 °C. Triethylamine (0.7 mL, 5.00 mmol) was then added dropwise over 10 min, followed by a solution of 1,2,4-triazole (0.414 g, 6.00 mmol) and triethylamine (0.8 mL, 6.00 mmol) in dry CH_3_CN over 30 min. The reaction mixture was stirred at room temperature for two days. It was then diluted with ethyl acetate (200 mL) and quenched with a NaH_2_PO_4_/Na_2_HPO_4_ buffer (pH = 7, 150.0 mL). The organic layer was washed with water and brine, dried over Na_2_SO_4_, and evaporated in vacuo to give a crude residue, which was purified by column chromatography (EtOAc:hexane 7:3) to give 5′-*O*-(*tert*-butyldiphenylsilyl)-3′-chloro-2′,3′-didehydro-2′,3′-dideoxy-4-(1*H*-1,2,4-triazol-1-yl)uridine as a white solid (0.884 mg, 80%). ^1^H-NMR (300 MHz, CDCl_3_): δ 9.24 (s, 1H, N-C*H*=N), 8.55 (d, 1H, *J* = 7.25 Hz, H-6), 8.07 (s, 1H, N-C*H*=N), 8.07–7.08 (m, 10H, Ar*H*), 7.09 (dd, 1H, *J* = 3.3, 1.5 Hz, H-1′), 6.28 (d, 1H, *J* = 7.25 Hz, H-5), 6.16 (t, 1H, *J* = 1.5 Hz, H-2′), 4.89–4.87 (m, 1H, H-4′), 4.13 (dd, 1H, *J* = 12.2, 1.6 Hz, H-5′), 4.11 (dd, 1H, *J* = 12.2, 2.0 Hz, H-5″), 1.11 (s, 9H, 3 × C*H*_3_); ^13^C-NMR (75 MHz, CDCl_3_): δ 154.0 (C=O), 147.8 (4-*C*N), 147.6 (N-*C*H=N), (6-C) 143.2, 141.8 (N-*C*H=N), 136.3 (3′-*C*(Cl)), 135.5 (Ar*C*), 135.0 (Ar*C*), 130.5 (Ar*C*), 130.4 (Ar*C*), 128.3 (Ar*C*), 128.2 (Ar*C*), 123.0 (2′-C), 94.9 (5-C), 90.3 (4′-*C*H), 87.5 (1′-*C*H), 62.5 (5′-*C*H_2_), 29.9 (*C*(CH_3_)_3_), 27.3 (3 × *C*H_3_); HRMS: C_27_H_28_ClN_5_O_3_Si [M + H^+^]^+^ Calc.: 534.1722, found: 534.1718. Following the general desilylation procedure, a solution of 5′-*O*-(*tert*-butyldiphenylsilyl)-3′-chloro-2′,3′-didehydro-2′,3′-dideoxy-4-(1*H*-1,2,4-triazol-1-yl)uridine (0.53 g, 1.00 mmol) in dry THF (2.00 mL) was reacted with TBAF (1.5 mL, 1.5 mmol) at −50 °C. After work-up, the resulting crude residue was purified by column chromatography (EtOAc) to give 3′-chloro-2′,3′-didehydro-2′,3′-dideoxy-4-(1*H*-1,2,4-triazol-1-yl)uridine as a white solid (0.221 mg, 75%). ^1^H-NMR (500 MHz, MeOD): δ 9.38 (s, 1H, N-C*H*=N), 8.87 (d, 1H, *J* = 7.2 Hz, H-6), 8.24 (s, 1H, N-C*H*=N), 7.12 (d, 1H, *J* = 7.2 Hz, H-5), 7.02 (dd, 1H, *J* = 2.5, 1.5 Hz, H-1′), 6.19 (t, 1H, *J* = 1.5 Hz, H-2′), 4.89 (br s, 1H, H-4′), 3.89 (dq, 2H, *J* = 12.8, 1.5 Hz, H-5′, H-5″); ^13^C-NMR (125 MHz, DMSO): δ 161.1 (4-C=N), 157.1 (2-C=O), 154.8 (N-*C*H=N), 150.5 (N-*C*H=N), 144.7 (6-C), 137.7 (3′-*C*(Cl)), 123.3 (2′-*C*H) 96.0 (5-C), 92.0 (4′-*C*H), 89.3 (1′-*C*H), 62.9 (5′-*C*H_2_); HRMS: C_11_H_10_ClN_5_O_3_ [M + H^+^]^+^ Calc.: 296.0544, found: 296.0542. To a stirred solution of 3′-chloro-2′,3′-didehydro-2′,3′-dideoxy-4-(1*H*-1,2,4-triazol-1-yl)uridine (0.295 g 0.01 mmol,) in EtOH (3.00 mL) at −20 °C was added a solution of NH_3_ in EtOH. The reaction mixture was allowed to warm to room temperature and stirred for 4 h. After removal of all the volatiles in vacuo, the crude residue was purified by column chromatography (EtOAc:EtOH 75:25) to give **23** as a pale green solid (0.194 g, 80%). ^1^H-NMR (600 MHz, MeOD): δ 8.12 (d, 1H, *J* = 7.5 Hz, H-6), 7.01 (dd, 1H, *J* = 3.3, 1.5 Hz, H-1′), 6.06 (t, 1H, *J* = 1.5, H-2′), 5.93 (d, 1H, *J* = 7.5 Hz, H-5), 4.87–4.77 (m, 1H, H-4′), 3.86 (dq, 2H, *J* = 12.8, 2.0 Hz, H-5′, H-5″); ^13^C-NMR (150 MHz, MeOD): δ 166.7 (4-C), 157.1 (C=O), 144.2 (6-C), 137.1 (3′-CCl), 123.7 (2′-C), 96.0 (5-C), 90.2 (1′-C), 88.4 (4′-*C*H), 61.2 (5′-*C*H_2_); HRMS: C_9_H_10_Cl_1_N_3_O_3_ [M + Na^+^]^+^ Calc.: 266.0303, found: 266.0308.

*5′-O-(tert-Butyldiphenylsilyl)-3′*,*3′-gem-dichloro-2′*,*3′-dideoxy-6-chloropurine* (**24**). To a stirred solution of 6-chloropurine (0.616 g, 4.00 mmol) in dry CH_3_CN (10 mL), *N*,*O*-bistrimethylsilylacetamide (1.09 mL, 4.50 mmol) was added, and then the reaction mixture was heated at 78 °C for 1 h. After cooling to room temperature, a solution of compound **22** (0.569 g, 1.00 mmol) in dry CH_3_CN (15 mL) was added. The reaction mixture was then cooled to −20 °C and TMSOTf (0.92 mL, 5.00 mmol) was added. The mixture was allowed to slowly warm to 20 °C and it was then heated at 78 °C and stirred for 5 h. It was then diluted with ethyl acetate and washed with saturated aq. NaHCO_3_ (50 mL), water (50 mL), and brine (50 mL). The organic layer was dried over Na_2_SO_4_, filtered, and concentrated in vacuo. The crude residue was purified by column chromatography (hexane:EtOAc 8.5:1.5 to 7:3) to give **24** as an anomeric mixture (0.448 g, 80%). This mixture was further purified by column chromatography to give 57% of **24a** (α*-*anomer) and 43% of **24b** (β*-*anomer). The unreacted excess of 6-chloropurine was recovered. Data for **24b**: ^1^H-NMR (300 MHz, CDCl_3_): δ 8.71 (s, 1H, H-2), 8.28 (s, 1H, H-8), 7.69–7.26 (m, 10H, Ar*H*), 6.54 (dd, 1H, *J* = 6.3, 5.3 Hz, H-1′), 4.55 (dd, 1H, *J* = 5.5, 3.4 Hz, H-4′), 4.20 (dd, 1H, *J* = 11.7, 3.3 Hz, H-5′), 4.09 (dd, 1H, *J* = 11.7, 5.5 Hz, H-5″), 3.44–3.42 (m, 2H, H-2′, H-2″), 1.06 (s, 9H, 3 × C*H*_3_); ^13^C-NMR (75 MHz, CDCl_3_): δ 152.4 (6-C), 151.8 (2-C), 151.3 (4-C), 144.0 (5-C), 135.9 (Ar*C*), 135.8 (Ar*C*), 133.0 (Ar*C*), 132.9 (Ar*C*), 130.2 (8-*C*), 128.0 (Ar*C*), 128.0 (Ar*C*), 90.1 (4′-CH), 85.5 (3′-*C*(Cl_2_)), 84.4 (1′-*C*H), 63.5 (5′-*C*H_2_), 52.3 (2′-*C*H_2_), 29.9 (*C*(CH_3_)_3_), 27.0 (3 × *C*H_3_); HRMS: C_26_H_27_Cl_3_N_4_O_2_Si [M + H^+^]^+^ Calc.: 561.1041, found: 561.1046.

*3′*,*3′-gem-Dichloro-2′*,*3′-dideoxyadenosine* (**3c**). Following the general desilylation procedure, **24b** (0.028 g, 0.05 mmol) was reacted with a solution of TBAF in THF (0.5 mL, 0.5 mmol) in dry THF (5 mL). After work-up, the resulting crude material was dissolved in dry MeOH (5 mL) and cooled to −20 °C. A saturated solution of NH_3_ in H_2_O (10 mL, 25%) was then added; the reaction mixture was allowed to warm to room temperature and stirred overnight. After removal of all the volatiles in vacuo, the crude residue was purified by column chromatography (MeOH:EtOAc 0:10 to 2.5:7.5) to give **3c** as a pale yellow solid (4 mg, 14%). Compounds **25** and **26** were also isolated as an oil (11%) and white solid (15%), respectively. Data for **3c**: ^1^H-NMR (600 MHz, DMSO-d_6_): δ 8.33 (s, 1H, H-2), 8.16 (s, 1H, H-8), 7.38 (s, 2H, N*H*_2_), 6.47 (t, 1H, *J* = 6.8 Hz, H-1′), 5.48 (t, 1H, *J* = 5.3 Hz, O*H*), 4.46 (dd, 1H, *J* = 5.0, 3.3 Hz, H-4′), 3.83 (ddd, 1H, *J* = 10.4, 5.0, 3.3 Hz, H-5′), 3.79 (ddd, 1H, *J* = 10.4, 5.3, 5.0 Hz, H-5″), 3.65 (dd, 1H, *J* = 14.6, 6.8 Hz, H-2′), 3.42 (dd, 1H, *J* = 14.6, 6.8 Hz, H-2″); ^13^C-NMR (150 MHz, DMSO-d_6_): δ 156.2 (6-C), 152.8 (2-C), 149.2 (4-C), 139.1 (8-C), 118.9 (5-*C*H), 89.7 (4′-*C*H), 87.0 (3′-*C*(Cl_2_)), 81.2 (1′-*C*H), 61.2 (5′-*C*H_2_), 49.9 (2′-*C*H_2_); HRMS: C_10_H_11_Cl_2_N_5_O_2_ [M + H^+^]^+^ Calc.: 304.0362, found: 304.0357. Data for **25**: ^1^H-NMR (600 MHz, DMSO-d_6_): δ 8.56 (s, 1H, H-2), 8.55 (s, 1H, H-8), 6.57 (dd, 1H, *J* = 7.1, 6.2 Hz, H-1′), 5.32 (t, 1H, *J* = 4.9 Hz, O*H*), 4.48 (dd, 1H, *J* = 5.3, 3.4 Hz, 1H, H-4′), 3.84 (ddd, 1H, *J* = 12.2, 5.0, 3.4 Hz, H-5′), 3.80 (ddd, 1H, *J* = 12.2, 5.3, 5.0 Hz, H-5″), 3.66 (dd, 1H, *J* = 14.6, 6.2 Hz, H-2′), 3.48 (dd, 1H, *J* = 14.6, 7.1 Hz, H-2″); ^13^C-NMR (150 MHz, DMSO-d_6_): δ 160.1 (6-C), 152.0 (2-C), 151.8 (4-C), 141.7 (8-C), 120.9 (5-*C*H), 89.7 (1′-*C*H), 86.7 (3′-*C*(Cl_2_)), 81.4 (4′-*C*H), 60.9 (5′-*C*H_2_), 50.0 (2′-*C*H_2_). Data for **26**: ^1^H-NMR (300 MHz, DMSO-d_6_): δ 8.23 (s, 1H, H-2), 8.15 (s, 1H, H-8), 7.29 (s, 2H, N*H*_2_), 6.96 (dd, 1H, *J* = 5.1, 1.6 Hz, H-1′), 6.37 (t, 1H, *J* = 1.6 Hz, H-2′), 5.06–5.03 (m, 1H, H-4′), 3.71 (dd, 1H, *J* = 12.7, 1.9 Hz, H-5″), 3.59 (dd, 1H, *J* = 12.7, 3.0 Hz, H-5′); ^13^C-NMR (75 MHz, DMSO-d_6_): δ 156.1 (6-C), 152.9 (2-C), 149.2 (4-C), 139.1 (8-C), 134.8 (3′-C), 122.0 (2′-*C*H), 119.0 (5-*C*H), 87.1 (1′-*C*H), 87.0 (4′-*C*H), 60.4 (5′-*C*H_2_); HRMS: C_10_H_10_ClN_5_O_2_ [M + H^+^]^+^ Calc.: 268.0595, found: 268.0587.

*5-O-(o-Toluoyl)-1*,*2-isopropylidene-*α*-d-xylofuranose* (**27**). d-xylose (10.0 g, 0.067 mol) was dissolved in acetone (260 mL) containing H_2_SO_4_ (0.66 M, 10.0 mL) and the solution was stirred for 30 min. A solution of Na_2_CO_3_ (13.0 g, 0.123 mol) in water (112 mL) was carefully added to the above cooled mixture, which was then stirred for further 2.5 h at 20 °C. Then, solid Na_2_CO_3_ (7.00 g, 0.066 mol) was added, Na_2_SO_4_ (22.3 g) was filtered off and washed with acetone, and the filtrate was evaporated in vacuo to afford a crude residue (14 g). This residue was resolubilized in a 9:1 mixture of EtOAc (270 mL) and methanol (30 mL), filtered, and evaporated in vacuo to give a yellow oil (12 g, 96%). This residue was dissolved in dry DMF (150 mL) under an inert atmosphere, cooled in an ice bath, and then *o*-toluoyl chloride (9.85 g, 8.31 mL, 0.064 mol) was added, followed by imidazole (4.35 g, 0.064 mol). The reaction mixture was allowed to warm to room temperature and stirred for 5 h. It was then diluted with EtOAc (300 mL) and washed with water (300 mL) and brine (100 mL). The organic layer was dried over Na_2_SO_4_, filtered, and evaporated in vacuo to give a crude residue, which was purified by column chromatography (hexane:EtOAc 7:3) to afford compound **27** (16.5 g, 84%) as a colorless oil. ^1^H-NMR (300 MHz, CDCl_3_): δ 7.92 (dd, 1H, *J* = 8.2, 1.6 Hz, Ar*H*), 7.41–7.20 (m, 3H, Ar*H*), 5.97 (d, *J* = 3.6 Hz, 1H, H-1′), 4.69 (dd, 1H, *J* = 13.4, 8.4 Hz, H-5′), 4.57 (d, 1H, *J* = 3.6 Hz, H-2′), 4.43 (dd, 1H, *J* = 13.4, 6.0 Hz, H-5″), 4.42 (ddd, 1H, *J* = 8.4, 6.0, 2.1 Hz, H-4′), 4.24 (d, 1H, *J* = 2.1, H-3′), 3.73 (s, 1H, O*H*), 2.57 (s, 3H, C*H*_3_), 1.49 (s, 3H, C*H*_3_), 1.30 (s, 3H, C*H*_3_); ^13^C-NMR (75 MHz, CDCl_3_): δ 168.2 (CO), 140.7 (Ar*C*), 132.7 (Ar*C*), 132.0 (Ar*C*), 131.1 (Ar*C*), 129.0 (Ar*C*), 126.0 (Ar*C*), 112.0 (O*C*O) 105.1 (1′-C), 85.4 (4′-C), 78.8 (3′-*C*H), 74.8 (2′-*C*H), 62.0 (5′-*C*H_2_), 27.0 (*C*H_3_), 26.4 (*C*H_3_), 22.0 (*C*H_3_); HRMS for C_16_H_20_O_6_ [M + Na^+^]^+^ Calc.: 331.1152, found: 331.1156.

*5-O-(o-Toluoyl)-1*,*2-diacetyl-3-deoxyxylofuranose* (**28**). Compound **27** (1.00 g, 3.10 mmol) and *N*,*N*-thiocarbonyldiimidazole (0.55 g, 3.10 mmol) were dissolved in dry DMF (20 mL) and then imidazole (0.034 g, 0.50 mmol) was added under a nitrogen atmosphere. The reaction mixture was stirred overnight, and it was then diluted with EtOAc (100 mL) and washed with water (100 mL) and brine (500 mL). The organic layer was dried over Na_2_SO_4_, filtered, and evaporated in vacuo to give a crude residue which was purified by column chromatography (hexane:EtOAc 7:3) to afford 5-*O*-(*o*-toluoyl)-3-*O*-imidazolylthiocarbonyl-1,2-isopropylidene-d-xylofuranose (1.10 g, 89%) as a pale yellow oil. ^1^H-NMR (300 MHz, CDCl_3_): δ 8.32 (s, 1H, NC*H*) 7.88–7.01 (m, 6H, Ar*H*, NC*H*), 6.06 (d, 1H, *J* = 3.8 Hz, H-1′), 5.99 (d, 1H, *J* = 2.9, H-3′), 4.81–4.76 (m, 1H, H-4′), 4.78 (d, 1H, *J* = 3.9 Hz, H-2′), 4.59–4.56 (m, 2H, H-5′, H-5″), 2.53 (s, 3H, Ar*CH*_3_), 1.57 (s, 3H, C*H*_3_), 1.35 (s, 3H, C*H*_3_); ^13^C-NMR (75 MHz, CDCl_3_): δ 182.2 (CS), 168.8 (CO), 140.7 (*C*=N), 137.2 (*C*=N), 132.6 (Ar*C*), 131.9 (Ar*C*), 131.5 (Ar*C*), 130.9 (Ar*C*), 126.0 (Ar*C*), 117.9 (Ar*C*), 112.9 (O*C*O), 105.1 (1′-C), 84.5 (4′-C), 83.0 (3′-*C*H), 76.9 (2′-*C*H), 61.1 (5′-*C*H_2_), 26.8 (*C*H_3_), 26.4 (*C*H_3_), 21.9 (*C*H_3_). 5-*O*-(*o*-Toluoyl)-3-*O*-imidazolylthiocarbonyl-1,2-isopropylidene-α-d-xylofuranose (1.05 g, 2.50 mmol) and AIBN (0.254 g, 1.50 mmol) were solubilized in toluene (50 mL). The resulting solution was stirred at 105 °C for 25 min, and then tri-*n*-butyltin hydride (0.87 mL, 3.00 mmol) was added dropwise over 1 h under a nitrogen atmosphere. The reaction mixture was stirred for an additional 2.5 h at 105 °C, and then it was cooled to room temperature and evaporated in vacuo. The crude residue was dissolved in EtOAc (200 mL) and washed with water (200 mL) and brine (100 mL). The organic layer was dried over Na_2_SO_4_, filtered, and evaporated in vacuo to give a crude product that was purified by column chromatography (hexane:EtOAc 4:1) to afford 5-*O*-(*o*-toluoyl)-1,2-isopropylidene-3-deoxyxylofuranose (0.50 g, 60%) as a colorless oil. ^1^H-NMR (300 MHz, CDCl_3_): δ 7.95–7.92 (m, 2H, Ar*H*), 7.42–7.37 (m, 2H, Ar*H*), 7.26–7.21 (m, 4H, Ar*H*), 5.96 (d, 1H, *J* = 3.8 Hz, H-1′), 5.87 (d, 1H, *J* = 3.7 Hz, H-1′), 4.79–4.76 (m, 1H, H-2′), 4.59 (dd, 1H, *J* = 10.5, 4.1 Hz, H-5′), 4.79–4.45 (m, 5H, H-4′, H-4′, H-5′, H-5″, H-5″), 2.21–2.15 (m, 1H, H-3′), 1.77 (ddd, 1H, *J* = 13.1, 10.7, 4.9 Hz, H-3″), 2.60 (s, 3H, Ar*CH*_3_), 2.59 (s, 3H, Ar*CH*_3_), 1.54 (s, 3H, C*H*_3_), 1.50 (s, 3H, C*H*_3_), 1.33 (s, 6H, 2 × C*H*_3_); ^13^C-NMR (75 MHz, CDCl_3_): δ 167.5 (CO), 140.5 (Ar*C*), 132.4 (Ar*C*), 132.3 (Ar*C*), 131.9 (Ar*C*), 131.0 (Ar*C*), 125.9 (Ar*C*), 112.0 (O*C*O), 105.5 (1′-C), 84.6 (4′-C), 76.0 (2′-*C*H), 62.5 (5′-*C*H_2_), 35.7 (3′-*C*H), 27.0 (*C*H_3_), 26.5 (*C*H_3_), 22.0 (*C*H_3_); HRMS for C_16_H_20_O_5_ [M + Na^+^]^+^ Calc.: 315.1203, found: 315.1195. To a round-bottom flask containing 5-*O*-(*o*-toluoyl)-1,2-isopropylidene-3-deoxyxylofuranose (0.292 g, 1.00 mmol) were added acetic acid (5.0 mL) and acetic anhydride (3.0 mL). The reaction mixture was then cooled to 0 °C and then H_2_SO_4_ (0.5 mL) was added the stirring was continued overnight. The reaction mixture was then diluted with EtOAc (50 mL) and washed with a saturated solution of NaHCO_3_ (200 mL), water (50 mL), and brine (50 mL). The organic layer was dried over Na_2_SO_4_, filtered, and evaporated in vacuo to give a crude residue that was purified by column chromatography (hexane:EtOAc 7:3) to afford compound **28** (0.32 g, 95%) as a colorless oil and anomeric mixture. ^1^H-NMR (300 MHz, CDCl_3_): δ 7.95 (d, 1H, *J* = 8.0 Hz, Ar*H*), 7.43–7.38 (m, 1H, Ar*H*), 7.27–7.21 (m, 2H, Ar*H*), 6.19 (s, 1H, H-1′), 5.23 (dd, 1H, *J* = 4.2, 1.5 Hz, H-2′), 4.73–4.67 (m, 1H, H-4′), 4.47 (dd, 1H, *J* = 11.8, 3.9 Hz, H-5′), 4.33 (dd, 1H, *J* = 11.8, 5.6 Hz, H-5″), 2.61 (s, 3H, Ar*CH*_3_), 2.25–2.20 (m, 2H, H-3′, H-3″), 2.09 (s, 3H, C*H*_3_), 1.96 (s, 3H, C*H*_3_); ^13^C-NMR (75 MHz, CDCl_3_): δ 170.2 (Ar*C*O), 169.5 (1′-*C*O), 167.3 (2′-*C*O), 140.7 (Ar*C*), 132.5 (Ar*C*), 132.0 (Ar*C*), 130.9 (Ar*C*), 125.9 (Ar*C*), 121.6 (Ar*C*), 99.6 (1′-*C*H), 78.9 (4′-*C*H), 76.9 (2′-*C*H), 66.1.0 (5′-*C*H_2_), 31.9 (3′-*C*H_2_), 22.0 (Ar*C*H_3_), 21.3 (CO*C*H_3_), 21.1 (CO*C*H_3_); HRMS for C_17_H_20_O_7_ [M − H]^−^ Calc.: 335.1136, found: 335.1121.

*5′-O-(o-Toluoyl)-2′-O-acetyl-3′-deoxythymidine* (**29a**). Following the general procedure for sugar-base condensation, a solution of thymine (0.302 g, 2.40 mmol) in dry CH_3_CN (10 mL) was reacted with *N*,*O*-bis(trimethylsilyl)acetamide (1.46 mL, 6.00 mmol), a solution of **28** (0.672 g, 2.00 mmol) in dry CH_3_CN (10 mL), and TMSOTf (0.46 mL, 2.50 mmol). After work-up, the crude residue was purified by silica gel column chromatography (hexane:EtOAc 3:2) to afford compound **29a** (0.803 g) as a white solid in quantitative yield. ^1^H-NMR (300 MHz, CDCl_3_): δ 8.76 (s, 1H, N*H*), 7.92–7.14 (m, 1H, Ar*H*), 7.45–7.40 (m, 1H, Ar*H*), 7.28–7.22 (m, 2H, Ar*H*), 7.14 (d, 1H, *J* = 1.2 Hz, H-6), 5.85 (d, 1H, *J* = 2.3 Hz, H-1′), 5.35 (dt, 1H, *J* = 9.5, 2.3 Hz, H-2′), 4.65 (dd, *J* = 12.6, 2.7 Hz, 1H, H-5′), 4.60 (dddd, *J* = 6.6, 5.6, 5.0, 2.7 Hz, 1H, H-4′), 4.47 (dd, 1H, *J* = 12.8, 5.0 Hz, H-5″), 2.60 (s, 3H, C*H*_3_), 2.40 (ddd, 1H, *J* = 14.0, 9.5, 6.6 Hz, H-3′), 2.19 (ddd, 1H, *J* = 14.0, 5.6, 2.2 Hz, H-3″), 2.12 (s, 3H, C*H*_3_), 1.65 (d, *J* = 1.1 Hz, 3H, 6-C*H*_3_); ^13^C-NMR (75 MHz, CDCl_3_): δ 170.3 (CO), 167.2 (CO), 163.7 (4-C), 150.2 (2-C), 140.8 (6-C), 135.8 (Ar*C*), 132.7 (Ar*C*), 132.5 (Ar*C*), 130.5 (Ar*C*), 126.2 (Ar*C*), 111.5 (5-*C*H), 91.3 (1′-*C*H), 77.9 (4′-*C*H), 77.6 (2′-*C*H), 64.6 (5′-*C*H_2_), 33.1 (3′-*C*H), 21.9 (*C*H_3_), 21.1 (*C*H_3_), 12.1 (6-*C*H_3_); HRMS for C_20_H_22_N_2_O_7_ [M + H^+^]^+^ Calc.: 403.1499, found: 403.1495.

*5′-O-(o-Toluoyl)-2′-O-acetyl-3′-deoxy-4-N-benzoylcytidine* (**29b**). Following the general procedure for sugar-base condensation, a solution of *N*^4^-benzoylcytosine (0.538 g, 2.40 mmol) in dry CH_3_CN (10 mL) was reacted with *N*,*O*-bis(trimethylsilyl)acetamide (0.73 mL, 3.00 mmol), a solution of **28** (0.672 g, 2.00 mmol) in dry CH_3_CN (10 mL), and TMSOTf (0.46 mL, 2.50 mmol). After work-up, the crude residue was purified by silica gel column chromatography (hexane:EtOAc 1:1) to afford product **29b** (0.677 g) as a white solid in 69% yield. The corresponding α-anomer was also isolated as a white solid (0.098 g, 10%). ^1^H-NMR (300 MHz, CDCl_3_): δ 8.00–7.99 (m, 1H, Ar*H*), 7.90–7.89 (m, 2H, Ar*H*), 7.79 (d, *J* = 7.8 Hz, 1H, H-6), 7.64–7.62 (m, 1H, Ar*H*), 7.54–7.52 (m, 2H, Ar*H*), 7.36–7.20 (m, 3H, Ar*H*), 6.38 (d, 1H, *J* = 7.8 Hz, H-5), 5.58–5.55 (m, 1H, H-4′), 5.51 (d, 1H, *J* = 4.7 Hz, H-1′), 4.42–4.40 (m, 1H, H-2′), 4.56 (dd, 1H, *J* = 12.0, 4.2 Hz, H-5′), 4.40 (dd, 1H, *J* = 12.0, 6.2 Hz, H-5″), 2.23–2.17 (m, 1H, H-3′), 2.10–2.06 (m, 1H, H-3″), 2.57 (s, 3H, C*H*_3_), 2.28 (s, 3H, C*H*_3_); ^13^C-NMR (75 MHz, CDCl_3_): δ 186.4 (NH*C*O), 167.7 (Ar*C*O), 166.6 (CH_3_*C*O), 162.2 (4-*C*N), 145.5 (2-*C*O), 140.6 (6-C), 133.3 (Ar*C*), 132.3 (Ar*C*), 131.7 (Ar*C*), 130.5 (Ar*C*), 129.1 (Ar*C*), 128.5 (Ar*C*), 127.5 (Ar*C*), 125.8 (Ar*C*), 97.3 (5-C), 92.0 (1′-*C*H), 73.8 (4′-C), 70.3 (2′-C), 62.2 (5′-*C*H_2_), 36.5 (3′-*C*H_2_), 21.9 (*C*H_3_), 21.7 (*C*H_3_); HRMS for C_26_H_25_N_3_O7 [M + H^+^]^+^ Calc.: 492.1765, found: 492.1763.

*5′-O-(o-Toluoyl)-2′-O-acetyl-3′-deoxy-6-chloropurine* (**29c**). Following the general procedure for sugar-base condensation, a solution of 6-chloropurine (0.371 g, 2.40 mmol) in dry CH_3_CN (10 mL) was reacted with *N*,*O*-bis(trimethylsilyl)acetamide (1.46 mL, 6.00 mmol), a solution of **28** (0.672 g, 2.00 mmol) in dry CH_3_CN (10 mL), and TMSOTf (0.46 mL, 2.50 mmol) After work-up, the crude residue was purified by silica gel column chromatography (hexane:EtOAc 3:2) to afford product **29c** (0.473 g) as a white solid in 55% yield. ^1^H-NMR (300 MHz, CDCl_3_): δ 8.65 (s, 1H, H-2), 8.24 (s, 1H, H-8), 7.78–7.16 (m, 4H, Ar*H*), 6.10 (d, 1H, *J* = 1.3 Hz, H-1′), 5.84 (dt, 1H, *J* = 6.1, 1.3 Hz, H-2′), 4.78 (dddd, 1H, *J* = 10.4, 5.6, 5.3, 3.1 Hz, H-4′), 4.66 (dd, 1H, *J* = 12.2, 3.1 Hz, H-5′), 4.50 (dd, 1H, *J* = 12.2, 5.3 Hz, H-5″), 2.85 (ddd, 1H, *J* = 14.0, 10.4, 6.2 Hz, H-3′), 2.54 (s, 3H, C*H*_3_), 2.34 (ddd, 1H, *J* = 14.0, 5.6, 1.3 Hz, H-3″), 2.16 (s, 3H, C*H*_3_); ^13^C-NMR (75 MHz, CDCl_3_): δ 170.4 (Ar*C*O), 167.1 (*C*O), 152.3 (6-C), 151.6 (2-C), 151.0 (4-C), 144.4 (8-C), 140.7 (Ar*C*), 132.7 (Ar*C*), 132.6 (Ar*C*), 132.1 (Ar*C*), 130.6 (Ar*C*), 128.8 (Ar*C*), 126.0 (5-*C*H), 90.9 (1′-*C*H), 79.4 (4′-*C*H), 78.0 (2′-*C*H), 64.6 (5′-*C*H_2_), 33.3 (3′-*C*H_2_), 21.9 (*C*H_3_), 21.1 (*C*H_3_); HRMS for C_20_H_19_Cl_1_N_4_O_5_ [M + H^+^]^+^ Calc.: 431.1116, found: 431.1115.

*5′-O-(tert-Butyldiphenylsilyl)-3′-deoxythymidine* (**30a**). To a solution of **29a** (0.402 g, 1.00 mmol) in dry MeOH (20 mL) was added K_2_CO_3_ (0.276 g, 2.00 mmol) and the reaction mixture was stirred at room temperature for 4 h. The solvent was then removed in vacuo and the resulting crude residue was purified by silica gel column chromatography (CH_2_Cl_2_:MeOH 9:1) to give 3′-deoxythymidine (0.241 g) as a white solid in quantitative yield. ^1^H-NMR (500 MHz, MeOD): δ 7.97 (d, 1H, *J* = 1.1 Hz, H-6), 5.68 (d, 1H, *J* = 1.8 Hz, H-1′), 4.41 (dddd, 1H, *J* = 9.9, 3.2, 2.6, 2.5 Hz, H-4′), 4.33–4.32 (dt, 1H, *J* = 5.6, 1.8 Hz, H-2′), 3.93 (dd, 1H, *J* = 12.4, 2.6 Hz, H-5′), 3.67 (dd, 1H, *J* = 12.4, 3.2 Hz, H-5″), 2.13 (ddd, 1H, *J* = 13.4, 9.9, 5.6 Hz, H-3′), 1.87 (ddd, 1H, *J* = 13.4, 5.6, 2.5 Hz, H-3″), 1.86 (d, 3H, *J* = 1.1 Hz, C*H*_3_); ^13^C-NMR (125 MHz, MeOD): δ 166.5 (4-C), 152.4 (2-C), 138.2 (6-C), 110.6 (5-*C*H), 93.6 (1′-*C*H), 82.5 (4′-*C*H), 77.0 (2′-*C*H), 63.1 (5′-*C*H_2_), 34.0 (3′-*C*H), 12.3 (6-*C*H_3_); HRMS for C_10_H_14_N_2_O_5_ [M + Na^+^]^+^ Calc.: 265.0795, found: 265.0796. To a stirred solution of 3′-deoxythymidine (0.242 g, 1.00 mmol) and imidazole (0.070 g, 1.00 mmol) in dry DMF (5.0 mL) was added TBDPSCl (0.275 g, 1.00 mmol) at −50 °C. The reaction mixture was allowed to warm to room temperature and stirred for 4 h. It was then diluted with EtOAc (100 mL) and washed with saturated aq. NaHCO_3_ (100 mL), water, and brine. The organic layer was dried over Na_2_SO_4_, filtered, and concentrated in vacuo. The crude residue was purified by silica gel column chromatography (hexane:EtOAc 3:2) to give product **30a** (0.456 g) as a white solid in 95% yield. ^1^H-NMR (600 MHz, CDCl_3_): ^1^H-NMR (600 MHz, CDCl_3_): δ 9.08 (s, 1H, NH), 7.66–7.64 (m, 4H, Ar*H*), 7.60 (d, 1H, *J* = 1.2 Hz, H-6), 7.45–7.37 (m, 6H, Ar*H*), 5.68 (d, 1H, *J* = 2.1 Hz, H-1′), 4.54–4.51 (m, 1H, H-4′), 4.46–4.44 (m, 1H, H-2′), 4.10 (dd, 1H, *J* = 11.8, 2.5 Hz, H-5′), 3.75 (dd, 1H, *J* = 11.8, 3.4 Hz, H-5″), 2.20 (ddd, 1H, *J* = 13.2, 8.6, 6.4 Hz, H-3′), 1.99 (ddd, 1H, *J* = 13.2, 6.1, 3.3 Hz, H-3′), 1.62 (d, *J* = 1.2 Hz, 3H, C*H*_3_), 1.08 (s, 9H, 3 × C*H*_3_); ^13^C-NMR (150 MHz, CDCl_3_): δ 163.7 (4-C), 150.9 (2-C), 135.5 (Ar*C*), 135.4 (Ar*C*), 135.1 (6-C), 133.0 (Ar*C*), 132.6 (Ar*C*), 130.0 (Ar*C*), 130.0 (Ar*C*), 127.9 (Ar*C*), 127.9 (Ar*C*), 110.3 (5-*C*H), 93.6 (1′-*C*H), 82.5 (4′-*C*H), 77.0 (2′-*C*H), 63.1 (5′-*C*H_2_), 34.0 (3′-*C*H), 29.6 (*C*(CH_3_)_3_), 26.9 (3 × *C*H_3_), 12.2 (6-*C*H_3_); HRMS for C_26_H_32_N_2_O_5_Si_1_ [M + H^+^]^+^ Calc.: 481.2153, found: 481.2154.

*5′-O-(tert-Butyldiphenylsilyl)-3′-deoxycytidine* (**30b**). To a solution of **29b** (0.491 g, 1.00 mmol) in dry MeOH (20 mL) was added K_2_CO_3_ (0.415 g, 3.00 mmol) and the reaction mixture was stirred at room temperature for 4 h. The solvent was then removed in vacuo and the resulting crude residue was purified by silica gel column chromatography (CH_2_Cl_2_:MeOH 9:1) to give 3′-deoxycytidine (0.225 g) as a white solid in quantitative yield. ^1^H-NMR (500 MHz, MeOD): δ 8.16 (d, 1H, *J* = 7.5 Hz, H-6), 5.86 (d, 1H, *J* = 7.5 Hz, H-5), 5.75 (s, 1H, H-1′), 4.45 (dddd, 1H, *J* = 10.7, 5.4, 3.4, 2.7 Hz, H-4′), 4.28 (dd, 1H, *J* = 5.2, 1.6 Hz, H-2′), 3.97 (dd, 1H, *J* = 12.4, 2.7 Hz, H-5′), 3.71 (dd, 1H, *J* = 12.4, 3.4 Hz, H-5′), 2.02 (ddd, 1H, *J* = 13.3, 10.7, 5.2 Hz, H-3′), 1.85 (ddd, 1H, *J* = 13.3, 5.4, 1.6 Hz, H-3″); ^13^C-NMR (150 MHz, MeOD): δ 167.8 (4-C), 158.4 (2-C), 142.6 (6-C), 118.6 (5-*C*H), 94.9 (1′-*C*H), 83.1 (4′-*C*H), 77.6 (2′-*C*H), 63.0 (5′-*C*H_2_), 33.5 (3′-*C*H_2_); HRMS for C_9_H_13_N_3_O_4_ [M + H^+^]^+^ Calc.: 228.0978, found: 228.0988. To a stirred solution of 3′-deoxycytidine (0.227 g, 1.00 mmol) and imidazole (0.070 g, 1.00 mmol) in dry DMF (5.0 mL) was added TBDPSCl (0.275 g, 1.00 mmol) at −50 °C. The reaction mixture was allowed to warm to room temperature and stirred for 4 h. It was then diluted with EtOAc (100 mL) and washed with saturated aq. NaHCO_3_ (100 mL), water, and brine. The organic layer was dried over Na_2_SO_4_, filtered, and concentrated in vacuo. The crude residue was purified by silica gel column chromatography (CH_2_Cl_2_:MeOH 9:1) to give compound **30b** (0.432 g) as a white solid in 93% yield. ^1^H-NMR (600 MHz, DMSO-d_6_): δ 7.80 (d, 1H, *J* = 7.4 Hz, H-6), 7.73–7.72 (m, 1H, Ar*H*), 7.65–7.62 (m, 3H, Ar*H*), 7.49–7.44 (m, 3H, Ar*H*), 7.35–7.33 (m, 1H, Ar*H*), 7.23–7.21 (m, 2H, Ar*H*), 7.01 (s, 2H, N*H*_2_), 5.72 (d, 1H, *J* = 1.1 Hz, H-1′), 5.51 (d, 1H, *J* = 7.4 Hz, H-5), 4.36 (dddd, 1H, *J* = 10.5, 3.5, 2.6, 1.5 Hz, H-4′), 4.12 (dt, 1H, *J* = 5.1, 1.1 Hz, H-2′), 4.02 (dd, 1H, *J* = 11.7, 2.6 Hz, H-5′), 3.72 (dd, 1H, *J* = 11.8, 3.5 Hz, H-5″), 3.50 (s, 1H, O*H*), 2.01 (ddd, 1H, *J* = 12.9, 5.2, 1.5 Hz, H-3′), 1.75 (ddd, 1H, *J* = 12.9, 10.5, 5.2 Hz, H-3″), 1.02 (s, 9H, 3 × C*H*_3_); ^13^C-NMR (150 MHz, DMSO-d_6_): δ 165.6 (4-C), 155.2 (2-C), 140.3 (6-C), 138.0 (Ar*C*), 135.2 (Ar*C*), 135.0 (Ar*C*), 132.7 (Ar*C*), 132.4 (Ar*C*), 131.1 (Ar*C*), 130.4 (Ar*C*), 130.1 (Ar*C*), 130.0 (Ar*C*), 129.7 (Ar*C*), 128.0 (Ar*C*), 125.5 (5-*C*H), 92.2 (1′-*C*H), 80.2 (4′-*C*H), 75.4 (2′-*C*H), 64.4 (5′-*C*H_2_), 33.1 (3′-*C*H), 29.6 (*C*(CH_3_)_3_), 26.7 (*C*H_3_); HRMS for C_25_H_31_N_3_O_4_Si_1_ [M + Na^+^]^+^ Calc.: 488.1976, found: 488.1976.

*5′-O-(tert-Butyldiphenylsilyl)-3′-deoxy-6-methoxy-adenosine* (**30c**). To a solution of **29c** (0.430 g, 1.00 mmol) in dry MeOH (20 mL) was added K_2_CO_3_ (0.415 g, 3.00 mmol) and the reaction mixture was stirred at room temperature for 4 h. The solvent was then removed in vacuo and the resulting crude residue was purified by silica gel column chromatography (CH_2_Cl_2_/MeOH 9:1) to give product 3′-deoxy-6-methoxy-adenosine (0.260 g) as a white solid in quantitative yield. ^1^H-NMR (500 MHz, MeOD): δ 8.62 (s, 1H, H-2), 8.52 (s, 1H, H-8), 6.08 (d, 1H, *J* = 2.2 Hz, H-1′), 4.74 (ddt, 1H, *J* = 5.8, 3.2, 2.2 Hz, H-2′), 4.56 (dddd, 1H, *J* = 8.7, 6.4, 3.5, 2.7 Hz, H-4′), 4.19 (s, 3H, OC*H*_3_), 3.95 (dd, *J* = 12.4, 2.7 Hz, 1H, H-5′), 3.70 (dd, 1H, *J* = 12.4, 3.5 Hz, H-5″), 2.40 (ddd, 1H, *J* = 13.4, 8.7, 5.8 Hz, H-3′), 2.07 (ddd, 1H, *J* = 13.4, 6.4, 3.2 Hz, H-3″); ^13^C-NMR (125 MHz, MeOD): δ 152.8 (6-C), 143.7 (2-C), 142.8 (4-C), 133.7 (8-C), 113.1 (5-CH), 84.2 (1′-*C*H), 73.4 (4′-*C*H), 67.4 (2′-*C*H), 54.5 (O*C*H_3_), 45.4 (5′-*C*H_2_), 45.4 (3′-*C*H_2_); HRMS for C_11_H_14_N_4_O_4_ [M + H^+^]^+^ Calc.: 267.1087, found: 267.1092. To a stirred mixture of 3′-deoxy-6-methoxy-adenosine (0.266 g, 1.00 mmol) and imidazole (0.070 g, 1.00 mmol) in dry DMF (5.0 mL) was added TBDPSCl (0.275 g, 1.00 mmol) at −50 °C. The reaction mixture was allowed to warm to room temperature and stirred for 4 h. It was then diluted with EtOAc (100 mL), and then washed with water and brine. The organic layer was dried over Na_2_SO_4_, filtered, and concentrated in vacuo. The crude residue was purified by silica gel column chromatography (hexane:EtOAc 7:3) to give compound **30c** (0.463 g) as a white solid in 92% yield. ^1^H-NMR (300 MHz, CDCl_3_): δ 8.50 (s, 1H, H-2), 8.31 (s, 1H, H-8), 7.66–7.60 (m, 4H, Ar*H*), 7.45–7.32 (m, 6H, Ar*H*), 5.98 (d, 1H, *J* = 2.7 Hz, H-1′), 5.13 (s, 1H, OH), 4.72 (m, 1H, H-2′), 4.64–4.59 (m, 1H, H-4′), 4.19 (s, 3H, OC*H*_3_), 4.00 (dd, 1H, *J* = 11.5, 3.1 Hz, H-5′), 3.73 (dd, 1H, *J* = 11.5, 3.5 Hz, H-5″), 2.39 (ddd, 1H, *J* = 13.1, 7.2, 5.7 Hz, H-3′), 2.13 (ddd, 1H, *J* = 13.1, 6.6, 4.5 Hz, H-3″), 1.02 (s, 9H, 3 × C*H*_3_); ^13^C-NMR (150 MHz, CDCl_3_): δ 161.4 (6-C), 152.0 (2-C), 151.0 (4-C), 140.5 (8-C), 135.8 (Ar*C*), 135.8 (Ar*C*), 133.0 (Ar*C*), 133.0 (Ar*C*), 130.1 (Ar*C*), 128.1 (Ar*C*), 128.0 (Ar*C*), 115.5 (5-*C*H), 93.2 (1′-*C*H), 81.7 (4′-*C*H), 76.7 (2′-*C*H), 65.2 (5′-*C*H_2_), 54.6 (O*C*H_3_), 33.4 (3′-*C*H_2_), 29.9 (*C*(CH_3_)_3_), 27.1 (*C*H_3_); HRMS: C_27_H_32_N_4_O_4_Si [M + H^+^]^+^ Calc.: 505.2265, found: 505.2269.

*5′-O-(tert-Butyldiphenylsilyl)-3′-deoxy-adenosine* (**30d**). A 50 mL round-bottomed flask was charged with **29c** (0.430 g, 1.00 mmol) and dry EtOH (10 mL) and the mixture was cooled to −20 °C. Then, a solution of NH_3_ in MeOH (10 mL, 7 N) was added and the mixture was stirred for 48 h. After removal of all the volatiles in vacuo, the resulting crude residue was purified by silica gel chromatography CH_2_Cl_2_/MeOH (7:3) to afford product 3′-deoxyadenosine (0.250 g) as a white solid in quantitative yield. ^1^H-NMR (600 MHz, D_2_O): δ 8.26 (s, 1H, H-2), 8.14 (s, 1H, H-8), 6.01 (d, 1H, *J* = 2.2 Hz, H-1′), 4.76 (m, 2H, H-2′, H-4′), 3.91 (dd, 2H, *J* = 12.6, 2.7 Hz, H-5′), 3.71 (dd, 1H, *J* = 12.6, 4.5 Hz, H-5″), 2.28 (ddd, 1H, *J* = 13.6, 8.7, 5.7 Hz, H-3′), 2.20 (ddd, 1H, *J* = 13.6, 6.6, 3.2 Hz, H-3″); ^13^C-NMR (150MHz, D_2_O): δ 155.3 (6-C), 152.3 (2-C), 148.0 (4-C), 139.6 (8-C), 118.6 (5-*C*H), 90.8 (1′-*C*H), 81.1 (4′-*C*H), 74.8 (2′-*C*H), 62.6 (5′-*C*H_2_), 32.9 (3′-CH_2_); HRMS for C_10_H_13_N_5_O_3_ [M + H^+^]^+^ Calc.: 252.1091, found: 252.1085. To a stirred mixture of 3′-deoxyadenosine (0.05 g, 0.25 mmol) and imidazole (0.070 g, 1.0 mmol) in anhydrous DMF (5 mL) was added TBDPSCl (0.275 g, 1.0 mmol) at −50 °C. The reaction mixture was allowed to warm to room temperature and stirred for 4 h. It was then diluted with EtOAc (100 mL) and washed with water and brine. The organic layer was dried over Na_2_SO_4_, filtered, and concentrated in vacuo. The crude residue was then purified by silica gel column chromatography (EtOAc) to afford compound **30d** (0.112 g, 92%) as a white solid. ^1^H-NMR (600 MHz, MeOD): δ 8.32 (s, 1H, H-2), 8.18 (s, 1H, H-8), 7.67–7.32 (m, 10H, Ar*H*), 6.02 (d, 1H, *J* = 1.5 Hz, H-1′), 4.71 (dt, 1H, *J* = 5.5, 1.6 Hz, H-2′), 4.57 (dddd, 1H, *J* = 8.9, 3.9, 2.8, 2.1 Hz, H-4′), 4.05 (dd, 1H, *J* = 11.6, 2.8 Hz, H-5′), 3.78 (dd, 1H, *J* = 11.7, 3.9 Hz, H-5″), 2.46 (ddd, 1H, *J* = 14.4, 8.9, 5.5 Hz, H-3′), 2.01 (ddd, 1H, *J* = 14.4, 5.6, 2.1 Hz, H-3″), 1.28 (s, 9H, 3 × C*H*_3_); ^13^C-NMR (150 MHz, MeOD): δ 157.3 (6-C), 153.8 (2-C), 150.0 (4-C), 140.3 (8-C), 136.7 (Ar*C*), 136.6 (Ar*C*), 134.2 (Ar*C*), 134.0 (Ar*C*), 131.0 (Ar*C*), 131.0 (Ar*C*), 128.8 (Ar*C*), 120.4 (5-*C*H), 93.2 (1′-*C*H), 82.7 (4′-*C*H), 77.0 (2′-*C*H), 66.0 (5′-*C*H_2_), 34.4 (3′-*C*H_2_), 31.6 (*C*CH_3_)_3_), 27.4 (CH_3_); HRMS for C_26_H_31_N_5_O_3_Si_1_ [M + H^+^]^+^ Calc.: 490.2268, found: 490.2273.

*5′-O-(tert-Butyldiphenylsilyl)-2′-O-o-toluoyl-4-N-o-toluoyl-3′-deoxycytidine* (**31a**). To a stirred solution of **30b** (0.93 g, 2.00 mmol) in dry DMF (30 mL), *ortho*-toluoyl chloride (0.386 g, 0.325 mL, 2.50 mmol) was added at 0 °C under an inert atmosphere. Next, imidazole (0.17 g, 2.50 mmol) was added and the mixture was stirred at room temperature for 3 h. It was then diluted with EtOAc (100 mL) and washed with water (100 mL) and brine (100 mL). The organic layer was dried over Na_2_SO_4_, filtered, and concentrated in vacuo. The crude residue was purified by silica gel column chromatography (hexane:EtOAc 7:3) to afford compound **31a** as a colorless oil (1.17 g, 84%). ^1^H-NMR (300 MHz, CDCl_3_): δ 8.52 (d, 1H, *J* = 7.5 Hz, H-6), 8.01–7.14 (m, 18H, Ar*H*), 6.26 (s, 1H, H-1′), 5.58 (d, 1H, *J* = 7.5 Hz, H-5), 5.27 (s, 1H, H-2′), 4.56–4.51 (m, 1H, H-4′), 4.27 (dd, 1H, *J* = 12.0, 2.0 Hz, H-5′), 3.79 (dd, 1H, *J* = 12.0, 2.5 Hz, H-5″), 2.62 (s, 3H, C*H*_3_) 2.51 (s, 3H, C*H*_3_) 2.14 (dd, 1H, *J* = 14.0, 4.9 Hz, H-3′), 2.13 (dd, *J* = 14.0, 4.9 Hz, 1H, H-3″), 1.15 (s, 9H, 3 × C*H*_3_); ^13^C-NMR (75 MHz, CDCl_3_): δ 171.1 (CO), 166.7 (CO), 163.2 (4-C), 154.9 (2-C), 145.0 (6-C), 138.3 (Ar*C*), 137.7 (Ar*C*), 135.8 (Ar*C*), 135.7 (Ar*C*), 132.1 (Ar*C*), 131.7 (Ar*C*), 131.2 (Ar*C*), 128.5 (Ar*C*), 128.3 (Ar*C*), 126.1 (Ar*C*), 125.8 (Ar*C*), 117.6 (5-*C*H), 91.3 (1′-*C*H), 82.0 (4′-*C*H), 79.2 (2′-*C*H), 63.6 (5′-*C*H_2_), 31.1 (3′-*C*H_2_), 29.9 (*C*(CH_3_)_3_), 27.2 (3 × *C*H_3_), 22.1 (Ar*C*H_3_), 22.1 (Ar*C*H_3_); HRMS: C_41_H_43_N_3_O_6_Si [M + Na^+^]^+^ Calc.: 724.2813, found: 724.2947.

*5′-O-(tert-Butyldiphenylsilyl)-2′-O-o-toluoyl-6-N-O-o-toluoyl-3′-deoxyadenosine* (**31b**). To a stirred solution of **30d** (0.489 g, 1.00 mmol) in dry pyridine (30 mL), *ortho*-toluoyl chloride (0.386 g, 0.325 mL, 2.5 mmol) was added at 0 °C under an inert atmosphere. The mixture was stirred at room temperature overnight. It was diluted with EtOAc (100 mL) and washed with water (100 mL) and brine (100 mL). The organic layer was dried over Na_2_SO_4_, filtered, and concentrated in vacuo. The crude residue was purified by silica gel column chromatography (hexane:EtOAc 7:3) to give compound **31b** as a colorless oil (0.580 g, 80%). ^1^H-NMR (600 MHz, CDCl_3_): δ 8.74 (s, 1H, H-2), 8.41 (s, 1H, H-8), 7.73–7.09 (m, 18H, Ar*H*), 5.96 (d, 1H, *J* = 3.1 Hz, H-1′), 4.78 (ddd, 1H, *J* = 5.8, 3.1, 2.4 Hz, H-2′), 4.62 (ddt, 1H, *J* = 6.9, 3.9, 3.5 Hz, H-4′), 3.95 (dd, 1H, *J* = 11.5, 3.5 Hz, H-5′), 3.73 (dd, 1H, *J* = 11.5, 3.9 Hz, H-5″), 2.49 (s, 6H, Ar*CH*_3_), 2.38 (ddd, 1H, *J* = 13.1, 6.9, 5.8 Hz, H-3′), 2.17 (ddd, 1H, *J* = 13.1, 6.9, 2.4 Hz, H-3″), 1.02 (s, 9H, 3 × C*H*_3_); ^13^C-NMR (150 MHz, CDCl_3_): δ 172.3 (CO), 152.2 (6-C), 151.8 (2-C), 151.6 (4-C), 143.1 (8-C), 139.2 (Ar*C*), 135.5 (Ar*C*), 134.9 (Ar*C*), 132.7 (Ar*C*), 132.7 (Ar*C*), 131.3 (Ar*C*), 131.3 (Ar*C*), 129.9 (Ar*C*), 129.3 (Ar*C*), 128.2 (Ar*C*), 127.7 (Ar*C*), 127.7 (Ar*C*), 125.4 (5-*C*H), 93.0 (1′-*C*H), 81.3 (4′-*C*H), 76.1 (2′-*C*H), 65.1 (5′-*C*H_2_), 33.5 (3′-*C*H_2_), 29.6 (*C*(CH_3_)_3_), 26.8 (3 × *C*H_3_), 19.9 (*C*H_3_), 19.1 (*C*H_3_); HRMS: C_42_H_43_N_5_O_5_Si [M + H^+^]^+^ Calc.: 726.3106, found: 726.3123.

*3′*,*5′-Bis-O-o-toluoyl-6-N-O-o-toluoyl-3′-deoxyadenosine* (**32**). To a stirred solution of 3′-deoxyadenosine (0.251 g, 1.00 mmol) in dry pyridine (30 mL), *o*-toluoyl chloride (0.539 g, 0.453 mL, 3.50 mmol) was added at 0 °C under an inert atmosphere and stirred overnight at room temperature. The mixture was diluted with EtOAc (100 mL) and washed with water (100 mL) and brine (100 mL). The organic layer was dried over Na_2_SO_4_, filtered, and evaporated in vacuo. The crude residue was purified by column chromatography (EtOAc:hexane 3:7) to afford **32** as a colorless oil (0.600 g, 99%). ^1^H-NMR (600 MHz, CDCl_3_): δ 8.49 (s, 1H, H-2), 8.10 (s, 1H, H-8), 7.99–7.19 (m, 12H, Ar*H*), 6.26 (d, 1H, *J* = 1.3 Hz, H-1′), 6.07 (dt, 1H, *J* = 6.0, 1.3 Hz, H-2′), 4.85 (dddd, 1H, *J* = 9.2, 5.6, 5.4, 3.1 Hz, H-4′), 4.68 (dd, 1H, *J* = 12.1, 3.1 Hz, H-5′), 4.53 (dd, 1H, *J* = 12.1, 5.4 Hz, H-5″), 3.18 (s, 3H, Ar*CH*_3_), 2.98 (ddd, 1H, *J* = 12.9, 9.2, 6.0 Hz, H-3′), 2.60 (s, 3H, Ar*CH*_3_), 2.56 (s, 3H, Ar*CH*_3_), 2.46 (ddd, 1H, *J* = 12.9, 5.6, 1.3 Hz, H-3″); ^13^C-NMR (150 MHz, CDCl_3_): δ 171.1 (NH*C*O), 166.9 (CO), 166.3 (CO), 161.0 (6-C), 152.2 (2-C), 151.0 (4-C), 141.1 (8-C), 140.8 (Ar*C*), 140.4 (Ar*C*), 132.7 (Ar*C*), 131.9 (Ar*C*), 131.7 (Ar*C*), 130.8 (Ar*C*), 130.4 (Ar*C*), 128.6 (Ar*C*), 128.0 (Ar*C*), 125.8 (Ar*C*), 125.7 (Ar*C*), 122.1 (5-*C*H), 90.4 (1′-*C*H), 78.9 (4′-*C*H), 78.1 (2′-*C*H), 64.6 (5′-*C*H_2_), 33.4 (3′-*C*H_2_), 21.9 (*C*H_3_), 21.8 (*C*H_3_), 21.6 (*C*H_3_).

*5′-O-(tert-Butyldiphenylsilyl)-2′-keto-3′-deoxythymidine* (**33a**). Following the general oxidation procedure, a solution of **30a** (0.480 g, 1.00 mmol) in CH_2_Cl_2_ (15 mL) was reacted with DMP (0.425 g, 1.00 mmol). After work-up, the crude residue was purified by silica gel column chromatography (hexane:EtOAc 7:3) to afford compound **33a** (0.476 g, 100%) as a white solid in quantitative yield. ^1^H-NMR (300 MHz, CDCl_3_): δ 10.2 (s, 1H, N*H*), 8.45–7.96 (m, 11H, Ar*H*, H-6), 6.02 (s, 1H, H-1′), 5.10 (dddd, 1H, *J* = 8.4, 7.3, 4.8, 3.6 Hz, H-4′), 4.67 (dd, 1H, *J* = 11.3, 3.6 Hz, H-5′), 4.59 (dd, 1H, *J* = 11.2, 4.8 Hz, H-5″), 3.60 (dd, 1H, *J* = 18.8, 8.4 Hz, H-3′), 3.22 (dd, 1H, *J* = 18.7, 7.3 Hz, H-3″), 2.44 (d, 3H, *J* = 1.0 Hz, C*H*_3_), 1.77 (s, 9H, 3 × C*H*_3_); ^13^C-NMR (75 MHz, CDCl_3_): δ 207.0 (2′-CO), 163.9 (4-C), 150.3 (2-C), 138.4 (6-C), 135.8 (Ar*C*), 135.7 (Ar*C*), 133.2 (Ar*C*), 133.2 (Ar*C*), 130.1 (Ar*C*), 128.0 (Ar*C*), 112.0 (5-*C*H), 85.9 (1′-*C*H), 76.2 (4′-*C*H), 65.4 (5′-*C*H_2_), 36.6 (3′-*C*H), 29.9 (*C*(CH_3_)), 27.1 (6-*C*H_3_) 12.3 (6-*C*H_3_); HRMS for C_26_H_30_N_2_O_5_Si_1_ [M + Na^+^]^+^ Calc.: 501.1816, found: 501.1817.

*5′-O-(tert-Butyldiphenylsilyl)-2′-keto-3′-deoxy-6-methoxy-adenosine* (**33b**). Following the general oxidation procedure, a solution of **30c** (0.504 g, 1.00 mmol) in CH_2_Cl_2_ (15 mL) was reacted with DMP (0.425 g, 1.00 mmol). After work-up, the crude residue was purified by silica gel column chromatography (hexane:EtOAc 7:3) to afford compound **33b** (0.500 g, 99%) as a white solid in quantitative yield. ^1^H-NMR (300 MHz, CDCl_3_): δ 8.40 (s, 1H, H-2), 7.96 (s, 1H, H-8), 7.62–7.29 (m, 10H, Ar*H*), 5.92 (s, 1H, H-1′), 4.61 (dddd, 1H, *J* = 8.7, 6.7, 4.4, 3.9 Hz, H-4′), 4.18 (s, 3H, OC*H*_3_), 4.03 (dd, 1H, *J* = 11.3, 3.9 Hz, H-5′), 3.90 (dd, 1H, *J* = 11.3, 4.4 Hz, H-5″), 3.24 (dd, 1H, *J* = 18.7, 8.7 Hz, H-3′), 2.79 (dd, 1H, *J* = 18.7, 6.7 Hz, H-3″), 1.03 (s, 9H, 3 × C*H*_3_); ^13^C-NMR (75 MHz, CDCl_3_): δ 206.9 (2′-CO), 161.4 (6-C), 152.7 (2-C), 151.7 (4-C), 141.5 (8-C), 135.8 (Ar*C*), 135.7 (Ar*C*), 133.0 (Ar*C*), 130.1 (Ar*C*), 128.0 (Ar*C*), 127.9 (Ar*C*), 122.0.0 (5-*C*H), 82.3 (1′-*C*H), 76.5 (4′-*C*H), 65.1 (5′-*C*H_2_), 54.6 (O*C*H_3_), 37.5 (3′-*C*H), 29.9 (*C*(CH_3_)), 27.0 (3 × *C*H_3_); HRMS for C_27_H_30_N_4_O_4_Si_1_ [M + H^+^]^+^ Calc.: 503.2108, found: 503.2102.

*5′-O-(tert-Butyldiphenylsilyl)-2′-keto-4-N-o-toluoyl-3′-deoxycytidine* (**33c**). To a solution of **31a** (0.701 g, 1.00 mmol) in dry THF (30 mL) at −78 °C under an inert atmosphere was added potassium *tert*-butoxide (0.112 g, 1.00 mmol), and the mixture was stirred for 1 h. It was then diluted with EtOAc (100 mL) and washed with water (100 mL) and brine (100 mL). The organic layer was dried over Na_2_SO_4_, filtered, and concentrated in vacuo. The crude residue was purified by silica gel column chromatography (EtOAc:hexane 1:1) to afford 5′-*O*-(*tert*-butyldiphenylsilyl)-4-*N*-*o*-toluoyl-3′-deoxycytidine as a colorless oil (0.466 g, 80%). ^1^H-NMR (300 MHz, CDCl_3_): δ 8.42 (d, 1H, *J* = 7.3 Hz, H-6), 7.66–7.26 (m, 14H, Ar*H*), 5.77 (s, 1H, H-1′), 4.59 (d, 1H, *J* = 7.3 Hz, H-5), 4.59 (m, 1H, H-2′), 4.45 (br s, 1H, OH), 4.15–4.08 (m, 2H, H-4′, H-5′), 3.73 (d, 1H, *J* = 9.9 Hz, H-5″), 2.31–2.17 (m, 2H, H-3′,H-3″), 2.50 (s, 3H, C*H*_3_), 1.25 (s, 9H, 3 × C*H*_3_); ^13^C-NMR (75 MHz, CDCl_3_): δ 168.9 (CO), 162.7 (4-C), 156.2 (2-C), 144.8 (6-C), 137.6 (Ar*C*), 135.8 (Ar*C*), 135.7 (Ar*C*), 135.7 (Ar*C*), 134.4 (Ar*C*), 133.0 (Ar*C*), 132.8 (Ar*C*), 131.9 (Ar*C*), 131.7 (Ar*C*), 130.3 (Ar*C*), 128.2 (Ar*C*), 127.4 (Ar*C*), 126.3 (Ar*C*), 96.5 (5-*C*H), 95.5 (1′-*C*H), 82.5 (4′-*C*H), 77.5 (2′-*C*H), 64.4 (5′-*C*H_2_), 32.7 (3′-*C*H_2_), 29.9 (*C*(CH_3_)_3_), 27.2 (3 × *C*H_3_), 20.3 (*C*H_3_); HRMS: C_33_H_37_N_3_O_5_Si [M + H^+^]^+^ Calc.: 584.2575, found: 584.2586. Following the general oxidation procedure, a solution of 5′-*O*-(*tert*-butyldiphenylsilyl)-4-*N*-*o*-toluoyl-3′-deoxycytidine (0.466 g, 0.80 mmol) in CH_2_Cl_2_ (15 mL) was reacted with DMP (0.425 g, 1.00 mmol). After work-up, the crude residue was purified by silica gel column chromatography (hexane:EtOAc 7:3) to afford compound **33c** (0.46 g, 100%) as a white solid in quantitative yield. ^1^H-NMR (300 MHz, CDCl_3_): 7.66–7.26 (m, 16H, Ar*H*, H-5, H-6), 5.40 (s, 1H, H-1′), 4.57–4.51 (m, 1H, H-4′), 3.99–3.97 (m, 2H, H-5′, H-5″), 3.03 (dd, 1H, *J* = 18.6, 8.1 Hz, H-3′), 2.63 (dd, 1H, *J* = 18.6, 7.6 Hz, H-3″), 2.51 (s, 3H, C*H*_3_), 1.25 (s, 9H, 3 × C*H*_3_); ^13^C-NMR (75 MHz, CDCl_3_): δ 206.0 (2′-CO), 163.6 (4-C), 154.6 (2-C), 148.2 (6-C), 137.9 (Ar*C*), 135.8 (Ar*C*), 135.8 (Ar*C*), 133.3 (Ar*C*), 132.0 (Ar*C*), 131.9 (Ar*C*), 130.1 (Ar*C*), 128.0 (Ar*C*), 128.0 (Ar*C*), 127.4 (Ar*C*), 126.4 (Ar*C*), 117.7 (5-*C*H), 97.4 (1′-*C*H), 87.7 (4′-*C*H), 66.0 (5′-*C*H_2_), 37.0 (3′-*C*H_2_), 29.9 (*C*(CH_3_)_3_), 27.1 (3 × *C*H_3_), 20.4 (*C*H_3_); HRMS: C_33_H_35_N_3_O_5_Si [M + H^+^]^+^ Calc.: 582.2418, found: 582.2429.

*5′-O-o-Toluoyl-2′-keto-6-N-o-toluoyl-3′-deoxyadenosine* (**33d**). To a stirred suspension of **32** (0.302 g, 0.50 mmol) in dry THF (10 mL) at −78 °C under an inert atmosphere was added potassium *tert*-butoxide (0.112 g, 1.00 mmol) and the mixture was stirred for 1 h. It was then diluted with EtOAc (100 mL) and washed with water (100 mL) and brine (100 mL). After work-up, the crude residue was purified by silica gel column chromatography (hexane:EtOAc 1:1) to afford 5′-*O*-*o*-toluoyl-6-*N*-*o*-toluoyl-3′-deoxyadenosine as a colorless oil (0.133 g, 55%). ^1^H-NMR (600 MHz, CDCl_3_): δ 9.34 (s, 1H, H-2), 8.94 (s, 1H, H-8), 8.61–7.73 (m, 8H, Ar*H*), 6.42 (d, 1H, *J* = 5.8 Hz, H-1′), 5.01 (m_)_, 1H, H-4′), 4.75 (m, 1H, H-2′), 4.64 (m, 2H, H-5′, H-5″), 3.65–3.55 (m, 1H, H-3′), 3.43–3.37 (m, 1H, H-3″), 3.27 (s, 3H, Ar*CH*_3_), 3.11 (s, 3H, Ar*CH*_3_); ^13^C-NMR (75 MHz, CDCl_3_): δ 172.5 (NH*C*O), 166.8 (CO), 153.1 (6-C), 152.6 (2-C), 151.8 (4-C), 143.2 (8-C), 140.8 (Ar*C*), 139.5 (Ar*C*), 135.9 (Ar*C*), 135.8 (Ar*C*), 133.0 (Ar*C*), 131.6 (Ar*C*), 130.2 (Ar*C*), 128.5 (Ar*C*), 128.2 (Ar*C*), 125.7 (5-*C*H), 85.8 (1′-*C*H), 84.8 (4′-*C*H), 75.1 (2′-*C*H), 64.2 (5′-*C*H_2_), 38.6 (3′-*C*H_2_), 27.2 (*C*H_3_), 20.2 (*C*H_3_). Following the general oxidation procedure, a solution of 5′-*O*-*o*-toluoyl-6-*N*-*o*-toluoyl-3′-deoxyadenosine (0.097 g, 0.20 mmol) in CH_2_Cl_2_ (10 mL) was reacted with DMP (0.106 g, 0.25 mmol). After work-up, the crude residue was purified by silica gel column chromatography (hexane:EtOAc 3:2) to afford compound **33d** (0.077 g, 80%) as a white solid. ^1^H-NMR (300 MHz, CDCl_3_): δ 8.40 (s, 1H, H-2), 7.94 (s, 1H, H-8), 8.05–7.18 (m, 8H, Ar*H*), 5.88 (s, 1H, H-1′), 4.89 (dddd, 1H, *J* = 9.2, 6.7, 5.4, 3.3 Hz, H-4′), 4.72 (dd, 1H, *J* = 12.0, 3.3 Hz, H-5′), 4.60 (dd, 1H, *J* = 12.1, 5.4 Hz, H-5″), 3.31 (dd, 1H, *J* = 18.5, 9.2 Hz, H-3′), 2.94 (dd, 1H, *J* = 18.6, 6.7 Hz, H-3″), 2.55 (s, 6H, C*H*_3_); ^13^C-NMR (75 MHz, CDCl_3_): δ 206.8 (2′-CO), 167.1 (CO), 161.5 (CO), 152.7 (6-C), 151.5 (2-C), 142.0 (4-C), 141.8 (8-C), 140.9 (Ar*C*), 132.7 (Ar*C*), 132.1 (Ar*C*), 130.9 (Ar*C*), 126.0 (Ar*C*), 122.0 (5-*C*H), 82.4 (1′-*C*H), 74.2 (4′-*C*H), 65.2 (5′-*C*H_2_), 37.9 (3′-*C*H_2_), 21.9 (*C*H_3_), 21.8 (*C*H_3_).

*2′*,*2′-gem-Dichloro-3′-deoxythymidine* (**4a**). Following the general chlorination procedure, a solution of compound **33a** (0.500 g, 1.04 mmol) in dry CH_2_Cl_2_ (20 mL) was reacted with PCl_5_ (0.822 g, 3.95 mmol) at −78 °C under an inert atmosphere. After work-up, the resulting crude residue was purified by column chromatography (hexane:EtOAc 4:1) to give 5′-*O*-(*tert*-butyldiphenylsilyl)-2′,2′-*gem*-dichloro-3′-deoxythymidine (0.283 g, 51%) as a major compound and 5′-*O*-(*tert*-butyldiphenylsilyl)-2′-chloro-2′,3′-didehydro-3′-deoxythymidine as a minor side product (0.049 g, 10%). ^1^H-NMR (500 MHz, CDCl_3_): δ 8.91 (s, 1H, N*H*), 7.66–7.37 (m, 11H, Ar*H*, H-6), 6.55 (s, 1H, H-1′), 4.37 (dddd, 1H, *J* = 12.4, 6.0, 3.2, 2.8 Hz, H-4′), 4.15 (dd, 1H, *J* = 14.3, 3.2 Hz, H-5′), 3.84 (dd, 1H, *J* = 14.3, 2.8 Hz, H-5″), 2.90 (dd, 1H, *J* = 16.7, 12.4 Hz, H-3′), 2.82 (dd, 1H, *J* = 16.7, 6.0 Hz, H-3″), 1.61 (s, 3H, C*H*_3_), 1.11 (s, 9H, 3 × C*H*_3_); ^13^C-NMR (125 MHz, CDCl_3_): δ 163.4 (4-C), 150.3 (2-C), 135.4 (6-C), 135.2 (Ar*C*), 134.2 (Ar*C*), 132.7 (Ar*C*), 132.3 (Ar*C*), 130.1 (Ar*C*), 131.1 (Ar*C*), 128.0 (Ar*C*), 111.0 (5-*C*H), 92.8 (1′-*C*H), 89.2 (2′-*C*(Cl)_2_), 78.5 (4′-*C*H), 62.8 (5′-*C*H_2_), 47.2 (3′-*C*H), 29.6 (*C*(CH_3_)), 26.9 (3 × *C*H_3_) 12.0 (6-*C*H_3_); HRMS: C_26_H_30_N_2_O_4_SiCl_2_ [M + H^+^]^+^ Calc.: 533.1424, found: 533.1428. Data for 5′-*O*-(*tert*-butyldiphenylsilyl)-2′-chloro-2′,3′-didehydro-3′-deoxythymidine: ^1^H-NMR (300 MHz, CDCl_3_): δ 8.23 (s, 1H, N*H*), 7.66–7.60 (m, 4H, Ar*H*), 7.46–7.344 (m, 6H, Ar*H*), 7.06 (d, 1H, *J* = 1.3 Hz, H-6), 6.89 (dd, 1H, *J* = 3.8, 1.7 Hz, H-1′), 6.30 (t, 1H, *J* = 1.7 Hz, H-3′), 4.95–4.93 (m, 1H, H-4′), 3.90–3.89 (m, 2H, H-5′, H-5″),1.46 (d, 3H, *J* = 1.3 Hz, C*H*_3_), 1.09 (s, 9H, 3 × C*H*_3_);HRMS: C_26_H_29_N_2_O_4_SiCl [M + Na^+^]^+^ Calc.: 519.1477, found: 519.1477. Following the general desilylation procedure, a solution of 5′-*O*-(*tert*-butyldiphenylsilyl)-2′,2′-*gem*-dichloro-3′-deoxythymidine (0.266 g, 0.50 mmol) in dry THF (10 mL) was reacted with TBAF (0.75 mL, 0.75 mmol). After work-up, the resulting crude residue was purified by column chromatography (EtOAc) to give **4a** as a white solid (0.132 g, 90%). ^1^H-NMR (600 MHz, MeOD): δ 8.18 (d, 1H, *J* = 2.4 Hz, H-6), 6.51 (s, 1H, H-1′), 4.44 (ddt, 1H, *J* = 10.8, 5.5, 2.4, Hz, H-4′), 4.06 (dd, 1H, *J* = 12.8, 2.4 Hz, H-5′), 3.76 (dd, 1H, *J* = 12.8, 2.4 Hz, H-5″), 2.86–2.85 (m, 2H, H-3′, H-3″), 1.88 (d, 3H, *J* = 2.3 Hz, C*H*_3_); ^13^C-NMR (150 MHz, MeOD): δ 166.1 (4-C), 152.5 (2-C), 136.8 (6-C), 111.4 (5-*C*H), 93.9 (1′-*C*H), 91.2 (2′-*C*(Cl)_2_), 81.6 (4′-*C*H), 61.1 (5′-*C*H_2_), 46.4 (3′-*C*H), 12.4 (6-CH_3_); HRMS: C_10_H_12_Cl_2_N_2_O_4_ [M + H^+^]^+^ Calc.: 295.0246, found: 295.0244. 

*2′*,*2′-gem-Dichloro-2′*,*3′-dideoxy-6-methoxy-adenosine* (**4b**). Following the general chlorination procedure, a solution of compound **33b** (0.500 g, 1.00 mmol) in dry CH_2_Cl_2_ (20 mL) was reacted with PCl_5_ (0.790 g, 3.80 mmol) at −78 °C under an inert atmosphere. After work-up, the resulting crude residue was purified by column chromatography (hexane:EtOAc 4:1) to give 5′-*O*-(*tert*-butyldiphenylsilyl)-2′,2′-*gem*-dichloro-2′,3′-dideoxy-6-methoxy-adenosine (0.359 g, 65%) as a pale yellow solid. ^1^H-NMR (300 MHz, CDCl_3_): δ 8.55 (s, 1H, H-2), 8.47 (s, 1H, H-8), 7.70–7.38 (m, 10H, Ar*H*), 6.67 (s, 1H, H-1′), 4.55 (dddd, 1H, *J* = 10.0, 5.3, 4.8, 3.2 Hz, H-4′), 4.20 (s, 3H, OC*H*_3_), 4.13 (dd, 1H, *J* = 11.9, 3.2 Hz, H-5′), 3.87 (dd, 1H, *J* = 8.5, 4.8 Hz, H-5″), 3.24 (dd, 1H, *J* = 13.9, 10.0 Hz, H-3′), 2.86 (dd, 1H, *J* = 13.9, 5.3 Hz, H-3″), 1.13 (s, 9H, 3 × C*H*_3_); ^13^C-NMR (75 MHz, CDCl_3_): δ 161.4 (6-C), 152.7 (2-C), 149.2 (4-C), 140.2 (8-C), 135.8 (Ar*C*), 135.7 (Ar*C*), 132.6 (Ar*C*), 130.3 (Ar*C*), 128.2 (Ar*C*), 128.0 (Ar*C*), 121.9 (5-*C*H), 93.0 (1′-*C*H), 89.0 (2′-*C*(Cl)_2_), 80.0 (4′-*C*H), 63.7 (5′-*C*H_2_), 54.5 (O*C*H_3_), 45.9 (3′-*C*H), 29.9 (*C*(CH_3_)), 27.0 (3 × *C*H_3_); HRMS: C_27_H_30_N_4_O_3_SiCl_2_ [M + H^+^]^+^ Calc.: 557.1536, found: 557.1219. Following the general desilylation procedure, a solution of 5′-*O*-(*tert*-butyldiphenylsilyl)-2′,2′-*gem*-dichloro-2′,3′-dideoxy-6-methoxy-adenosine (0.278 g, 0.50 mmol) in dry THF (10 mL) was reacted with TBAF (0.75 mL, 0.75 mmol). After work-up, the resulting crude residue was purified by column chromatography (EtOAc) to give compound **4b** as a white solid (151 mg, 95%). ^1^H-NMR (600 MHz, MeOD): δ 8.95 (s, 1H, H-2), 8.58 (s, 1H, H-8), 6.77 (s, 1H, H-1′), 4.62 (dddd, 1H, *J* = 10.5, 5.1, 2.9, 2.6 Hz, H-4′), 4.21 (s, 3H, OC*H*_3_), 4.09 (dd, 1H, *J* = 12.6, 2.6 Hz, H-5′), 3.87 (dd, 1H, *J* = 12.6, 2.9 Hz, H-5″), 3.20 (dd, 1H, *J* = 14.1, 10.5 Hz, H-3′), 2.98 (dd, 1H, *J* = 14.1, 5.1 Hz, H-3″); ^13^C-NMR (150 MHz, MeOD): δ 162.4 (6-C), 153.7 (2-C), 153.1 (4-C), 142.4 (8-C), 121.8 (5-*C*H), 94.2 (1′-*C*H), 90.2 (2′-*C*(Cl)_2_), 82.4 (4′-*C*H), 61.7 (5′-*C*H_2_), 54.9 (O*C*H_3_), 45.7 (3′-*C*H); HRMS: C_11_H_12_Cl_2_N_4_O_3_ [M + H^+^]^+^ Calc.: 319.0359, found: 319.0361. 

*2′*,*2′-gem-Dichloro-4-N-o-toluoyl-2′*,*3′-dideoxycytidine* (**34**). Following the general chlorination procedure, a solution of compound **33c** (0.46 g, 0.80 mmol) in dry CH_2_Cl_2_ (20 mL) was reacted with PCl_5_ (0.632 g, 3.04 mmol) at −78 °C under an inert atmosphere. After work-up, the resulting crude residue was purified by column chromatography (hexane:EtOAc 4:1) to give 5′-*O*-(*tert*-butyldiphenylsilyl)-2′,2′-*gem*-dichloro-4-*N*-*o*-toluoyl-2′,3′-dideoxycytidine (0.350 g, 69%) as a pale yellow solid. ^1^H-NMR (300 MHz, CDCl_3_): 8.40 (d, 1H, *J* = 7.5 Hz, H-6), 7.68–7.26 (m, 15H, Ar*H*, H-5), 6.67 (s, 1H, H-1′), 4.45 (ddt, 1H, *J* = 10.8, 4.7, 2.3 Hz, H-4′), 4.24 (dd, 1H, *J* = 12.2, 2.3 Hz, H-5′), 3.80 (dd, 1H, *J* = 12.2, 2.3 Hz, H-5″), 2.94 (dd, 1H, *J* = 13.4, 10.8 Hz, H-3′), 2.76 (dd, 1H, *J* = 13.4, 4.7 Hz, H-3″), 2.52 (s, 3H, C*H*_3_), 1.15 (s, 9H, 3 × C*H*_3_); ^13^C-NMR (75 MHz, CDCl_3_): δ 162.7 (4-C), 155.3 (2-C), 144.1 (6-C), 137.7 (Ar*C*), 135.8 (Ar*C*), 135.6 (Ar*C*), 134.3 (Ar*C*), 132.6 (Ar*C*), 132.3 (Ar*C*), 132.0 (Ar*C*), 131.8 (Ar*C*), 130.6 (Ar*C*), 130.5 (Ar*C*), 128.4 (Ar*C*), 128.3 (Ar*C*), 127.2 (Ar*C*), 126.4 (Ar*C*), 96.8 (5-*C*H), 93.4 (1′-*C*H), 89.0 (2′-C(Cl)_2_), 79.7 (4′-*C*H), 62.7 (5′-*C*H_2_), 46.0 (3′-*C*H_2_), 29.5 (*C*(CH_3_)_3_), 27.2 (3 × *C*H_3_), 20.4 (*C*H_3_); HRMS: C_33_H_35_Cl_2_N_3_O_4_Si [M + H^+^]^+^ Calc.: 636.1846, found: 636.1848. Following the general desilylation procedure, a solution of 5′-*O*-(*tert*-butyldiphenylsilyl)-2′,2′-*gem*-dichloro-4-*N*-*o*-toluoyl-2′,3′-dideoxycytidine (0.350 g, 0.55 mmol) in dry THF (10 mL) was reacted with TBAF (0.82 mL, 0.82 mmol). After work-up, the resulting crude residue was purified by column chromatography (EtOAc) to give compound **34** as a white solid (0.217 g, 100%). ^1^H-NMR (600 MHz, MeOD): 8.76 (d, 1H, *J* = 7.5 Hz, H-6), 7.59 (d, 1H, *J* = 7.5 Hz, H-5), 7.53–7.29 (m, 4H, Ar*H*), 6.67 (s, 1H, H-1′), 4.53 (ddt, 1H, *J* = 9.7, 6.2, 2.5, Hz, H-4′), 4.06 (dd, 1H, *J* = 12.8, 2.5 Hz, H-5′), 3.79 (dd, 1H, *J* = 12.8, 2.5 Hz, H-5″), 2.88–2.86 (m, 2H, H-3′, H-3′), 2.46 (s, 3H, C*H*_3_); ^13^C-NMR (150 MHz, MeOD): δ 171.7 (CO), 165.0 (4-C), 158.0 (2-C), 145.7 (6-C), 138.0 (Ar*C*), 136.2 (Ar*C*), 132.2 (Ar*C*), 132.2 (Ar*C*), 128.6 (Ar*C*), 126.9 (Ar*C*), 98.3 (5-*C*H), 94.8 (1′-*C*H), 90.6 (2′-C(Cl)_2_), 82.0 (4′-*C*H), 61.1 (5′-*C*H_2_), 46.4 (3′-*C*H_2_), 19.9 (*C*H_3_); HRMS: C_17_H_17_Cl_2_N_3_O_4_ [M + H^+^]^+^ Calc.: 398.0668, found: 398.0665.

*5′-O-o-Toluoyl-2′*,*2′-gem-dichloro-6-N-o-toluoyl-2′*,*3′-dideoxyadenosine* (**35**). Following the general chlorination procedure, a solution of compound **33d** (0.048 g, 0.10 mmol) in dry CH_2_Cl_2_ (5 mL) was reacted with PCl_5_ (0.079 g, 0.38 mmol) at −78 °C under an inert atmosphere. After work-up, the resulting crude residue was purified by column chromatography (hexane:EtOAc 7.5:2.5) to give compound **35** (0.037 g, 70%) as a pale yellow solid. ^1^H-NMR (600 MHz, MeOD): δ 9.08 (s, 1H, H-2), 8.76 (s, 1H, H-8), 7.67–7.08 (m, 8H, Ar*H*), 6.76 (s, 1H, H-1′), 4.60 (dddd, 1H, *J* = 10.5, 5.2, 3.0, 2.7 Hz, H-4′), 4.06 (dd, 1H, *J* = 12.6, 2.7 Hz, H-5′), 3.84 (dd, 1H, *J* = 12.6, 3.0 Hz, H-5″), 3.14 (dd, 1H, *J* = 14.2, 10.5 Hz, H-3′), 2.95 (dd, 1H, *J* = 14.2, 5.2 Hz, H-3″), 2.45 (s, 6H, 2 × C*H*_3_); ^13^C-NMR (150 MHz, MeOD): δ 173.5 (NH*C*O), 170.2 (CO), 154.6 (6-C), 153.5 (2-C), 152.5 (4-C), 145.5 (8-C), 140.1 (Ar*C*), 136.1 (Ar*C*), 132.6 (Ar*C*), 132.3 (Ar*C*), 129.9 (Ar*C*), 129.1 (Ar*C*), 126.5 (Ar*C*), 126.5 (5-*C*H), 94.2 (1′-*C*H), 90.6 (2′- C(Cl)_2_), 82.5 (4′-*C*H), 61.5 (5′-*C*H_2_), 45.7 (3′-*C*H_2_), 20.1 (*C*H_3_), 14.4 (*C*H_3_); HRMS: C_26_H_23_Cl_2_N_5_O_4_ [M + H^+^]^+^ Calc.: 540.1199, found: 540.1211.

*2′*,*2′-gem-Dichloro-2′*,*3′-dideoxycytidine* (**4c**). To a stirred solution of **34** (0.050 g, 0.125 mmol) in EtOH (3.0 mL) at −20 °C was added a saturated solution of NH_3_ in EtOH. The reaction mixture was then stirred for 2 days at room temperature. After removal of all the volatiles in vacuo, the crude residue was purified by silica gel column chromatography (EtOAc:MeOH 7.5:2.5) to give **4c** as a white solid (0.031 g, 89%). HRMS: ^1^H-NMR (600 MHz, MeOD): 8.21 (d, 1H, *J* = 7.6 Hz, H-6), 6.63 (s, 1H, H-1′), 5.91 (d, 1H, *J* = 7.6 Hz, H-5), 4.43 (ddt, 1H, *J* = 10.4, 5.1, 2.6 Hz, H-4′), 4.02 (dd, 1H, *J* = 12.8, 2.6 Hz, H-5′), 3.76 (dd, 1H, *J* = 12.7, 2.7 Hz, H-5″), 2.84 (dd, 1H, *J* = 13.7, 10.4 Hz, H-3′), 2.79 (dd, 1H, *J* = 13.8, 5.1 Hz, H-3′); ^13^C-NMR (150 MHz, MeOD): δ 167.6 (4-C), 158.2 (2-C), 141.7 (6-C), 96.1 (5-*C*H), 94.5 (1′-*C*H), 91.2 (2′-(*C*Cl)_2_), 81.2 (4′-*C*H), 61.3 (5′-*C*H_2_), 46.7 (3′-*C*H_2_); C_9_H_11_Cl_2_N_3_O_3_ [M + H^+^]^+^ Calc.: 280.0250, found: 280.0250.

*2′*,*2′-gem-Dichloro-2′*,*3′-dideoxyadenosine* (**4d**). To a stirred solution of **35** (0.037 g, 0.068 mmol) in dry MeOH (5.0 mL) was added K_2_CO_3_ (0.138 g, 0.10 mmol), and then the reaction mixture was stirred for 48 h. After removal of all the volatiles, the crude residue was purified by silica gel column chromatography (MeOH:CHCl_3_ 1.5:8.5 to 2.5:7.5) to give **4d** as a white solid (0.0092 mg, 45%). ^1^H-NMR (300 MHz, DMSO-d_6_): δ 8.88 (s, 1H, H-2), 8.62 (s, 1H, H-8), 6.70 (s, 1H, H-1′), 5.52 (s, 1H, O*H*), 4.51 (dddd, 1H, *J* = 8.7, 7.7, 3.9, 3.2, H-4′), 3.92 (dd, 1H, *J* = 12.5, 8.7 Hz, H-5′), 3.76 (dd, 1H, *J* = 12.5, 3.2 Hz, H-5′), 3.17 (dd, 1H, *J* = 11.1, 7.7 Hz, H-3′), 3.06 (dd, 1H, *J* = 11.1, 3.9 Hz, H-3″); ^13^C-NMR (75 MHz, DMSO-d_6_): δ 160.6 (6-C), 152.3 (2-C), 151.9 (4-C), 141.0 (8-C), 120.7 (5-*C*H), 92.1 (1′-*C*H), 89.8 (2′-*C*(*C*Cl)_2_), 81.0 (4′-*C*H), 60.5 (5′-*C*H_2_), 44.4 (3′-*C*H_2_); HRMS: C_10_H_11_Cl_2_N_5_O_2_ [M + NH_4_^+^]^+^ Calc.: 321.0395, found: 321.0328.

## 4. Conclusions

In this study, a new method for the preparation of two different sets of sugar chlorinated nucleosides starting from 2′-and 3′-ketonucleoside intermediates was developed. The reaction of 3′-keto-2′,3′-dideoxynucleosides in the presence of phosphorus pentachloride afforded the corresponding 3′,3′-*gem*-dichloride derivatives in moderate yields. An elimination reaction was found to be a significant side process leading to the concomitant formation of vinyl 3′-monochlorinated products. The method was extended to the chemoselective formation of 2′,2′-*gem*-dichloro-2′,3′-dideoxynucleosides in good yields under the same reaction conditions. This work contributes to the development of general synthetic strategies for the synthesis of modified nucleosides as well as structure-activity relationship studies on such compounds as potential antiviral agents.

## Supplementary Materials

The following are available online. Physical and spectroscopic data of the relevant compounds.
