# Supplementary material for: Phosphorus Pentachloride Promoted gem-Dichlorination of 2′- and 3′-Deoxynucleosides
Source: Molecules. 2018 Jun 15;23(6):1457. doi: 10.3390/molecules23061457 (PMC6100136; doi:10.3390/molecules23061457)

Molecules 2018, 23, 1457

Phosphorus Pentachloride Promoted *gem*-3 Dichlorination of 2′- and 3′-Deoxynucleosides

Fábio da Paixão Soares, Elisabetta Groaz and Piet Herdewijn*

KU Leuven, Rega Institute for Medical Research, Medicinal Chemistry, Herestraat 49, 3000 Leuven, Belgium; fabio.dapaixaosoares@student.kuleuven.be (F.P.S.); elisabetta.groaz@kuleuven.be (E.G.)

**Correspondence: piet.herdewijn@kuleuven.be (P.H.); Tel.: +32-16-322-657*

Supporting Information

^{1}H NMR (600 MHz, DMSO-*d6*) spectrum of Compound **3a**.

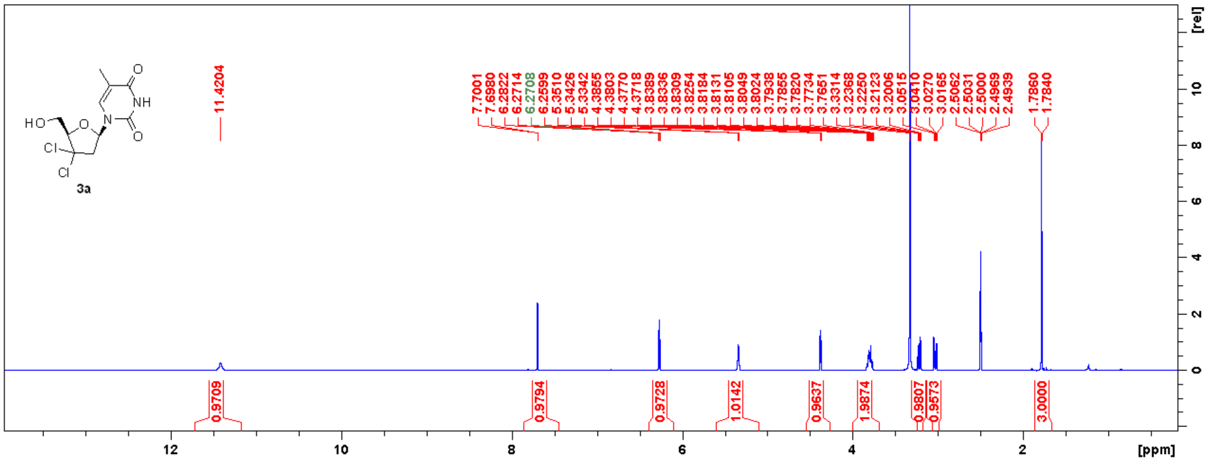

^{13}C NMR (150 MHz, DMSO-*d6*) spectrum of Compound **3a**.

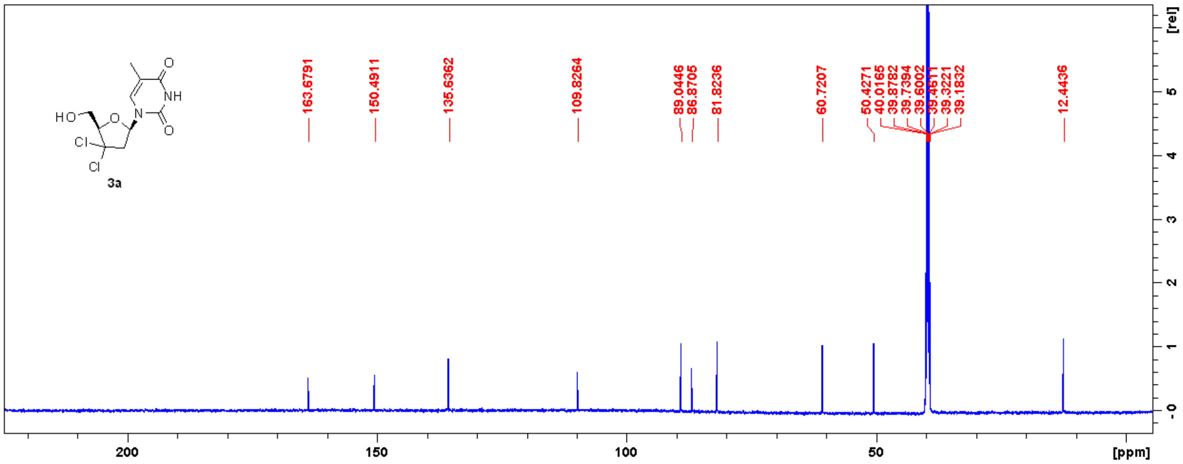

^{1}H NMR (600 MHz, DMSO-*d6*) spectrum of Compound **18**.

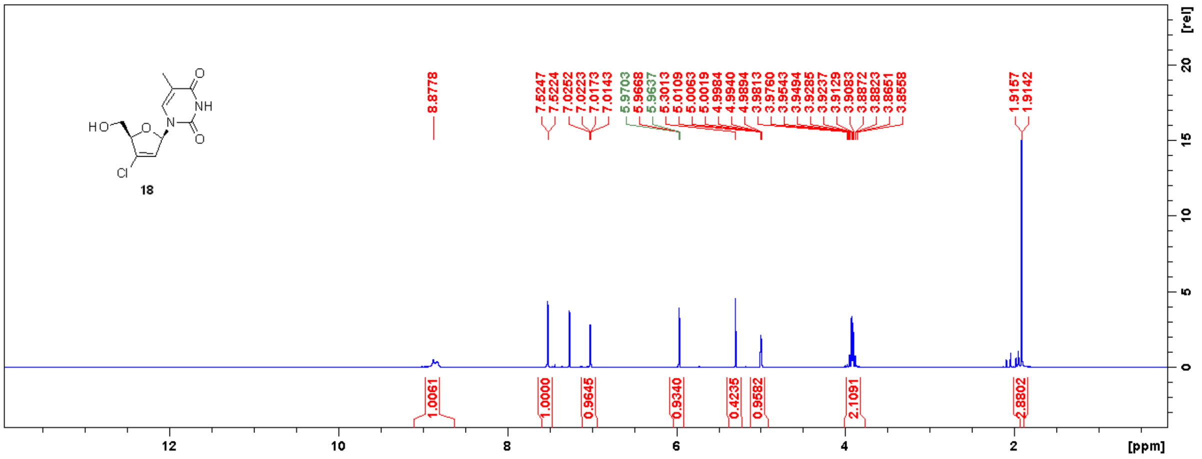

^{13}C NMR (150 MHz, DMSO-*d6*) spectrum of Compound **18**.

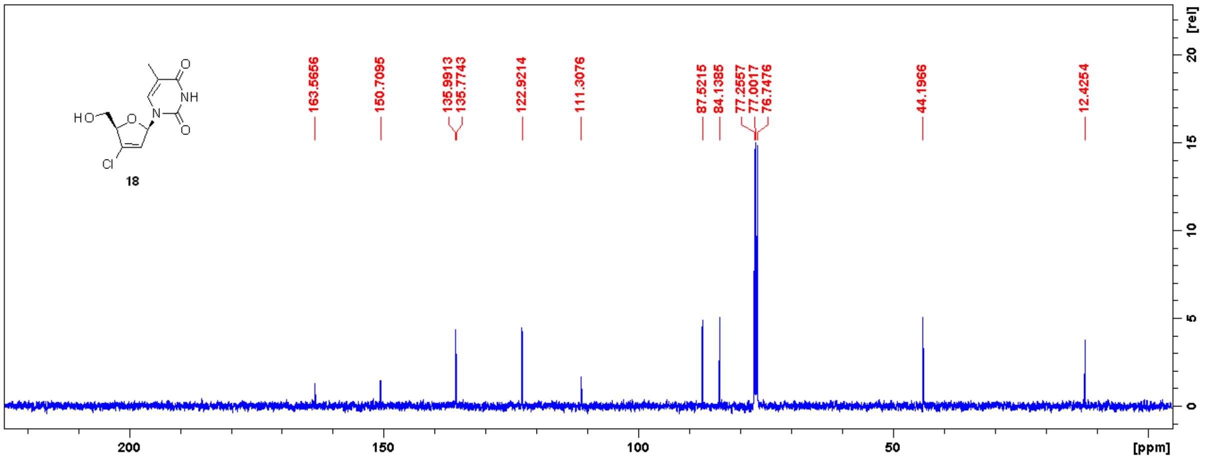

^{1}H NMR (600 MHz, DMSO-*d6*) spectrum of Compound **3b**.

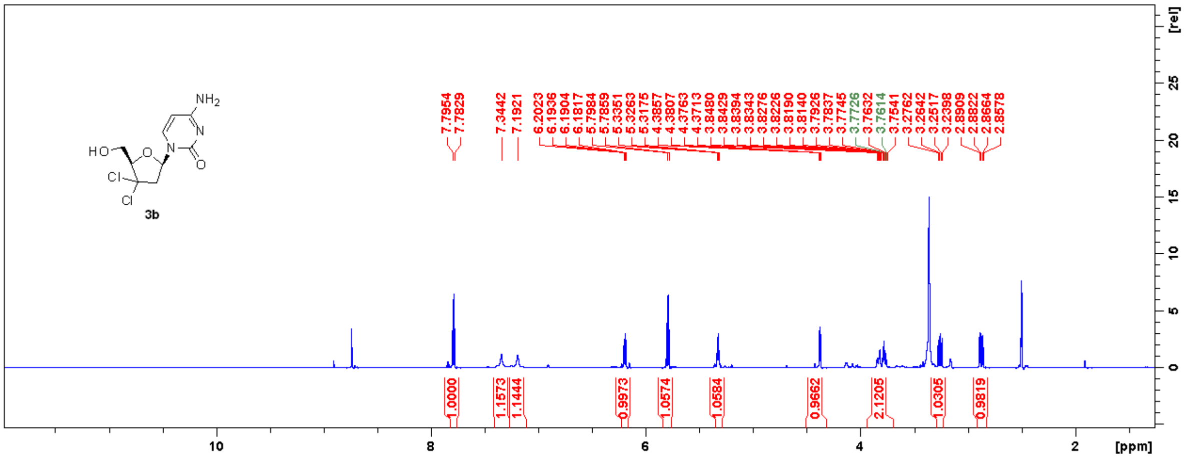

^{13}C NMR (150 MHz, DMSO-*d6*) spectrum of Compound **3b**.

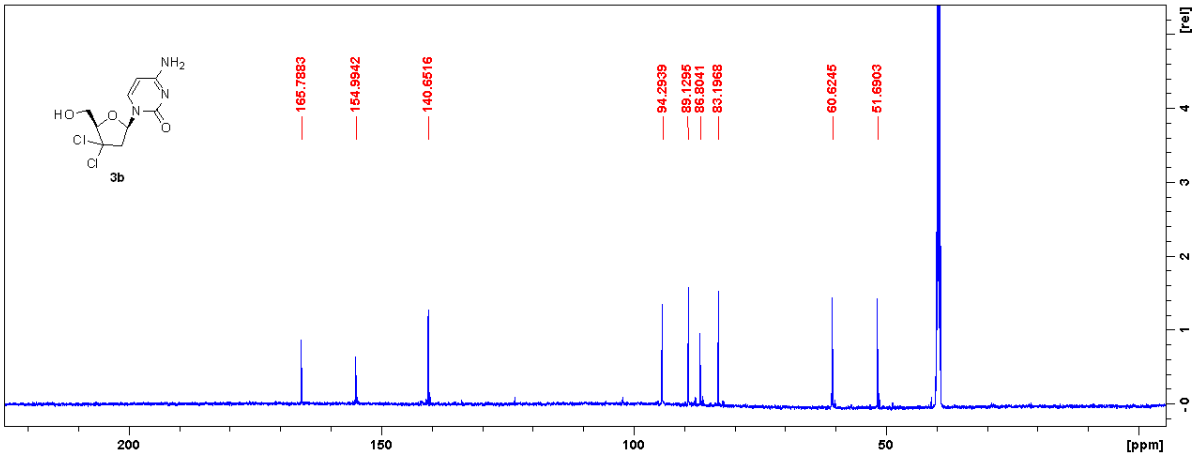

^{1}H NMR (600 MHz, MeOD-*d4*) spectrum of Compound **23**.

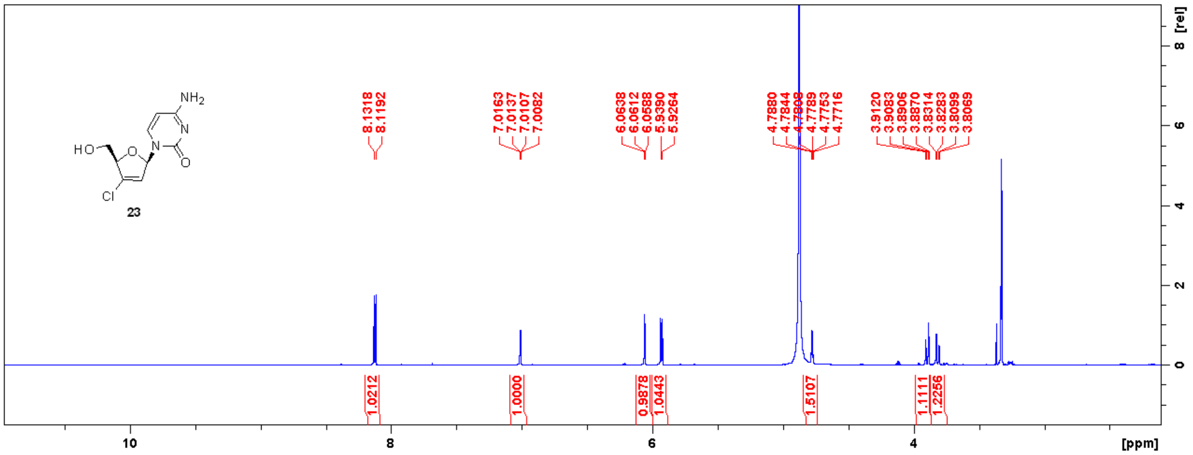

^{13}C NMR (150 MHz, MeOD-*d4*) spectrum of Compound **23**.

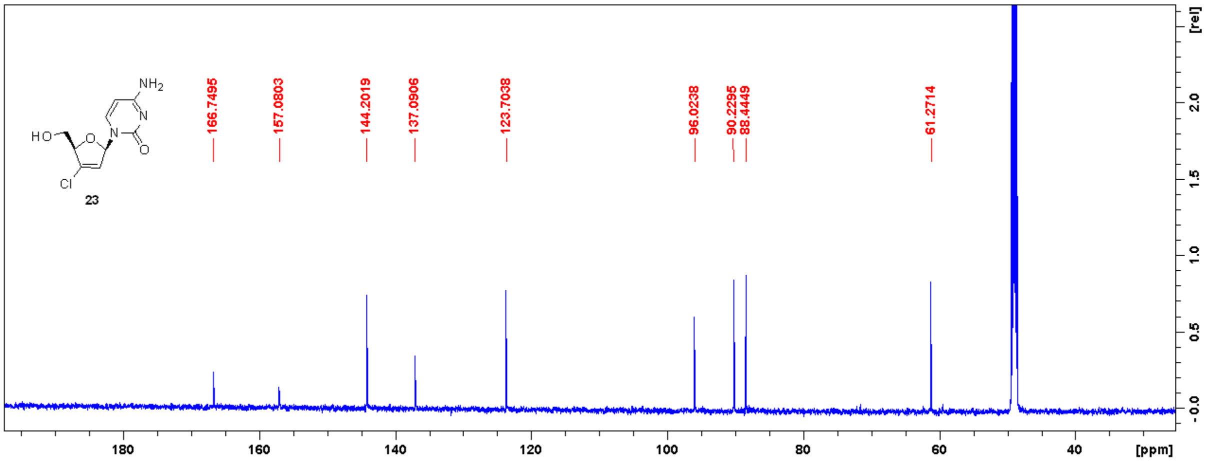

^{1}H NMR (600 MHz, DMSO-*d6*) spectrum of Compound **3c**.

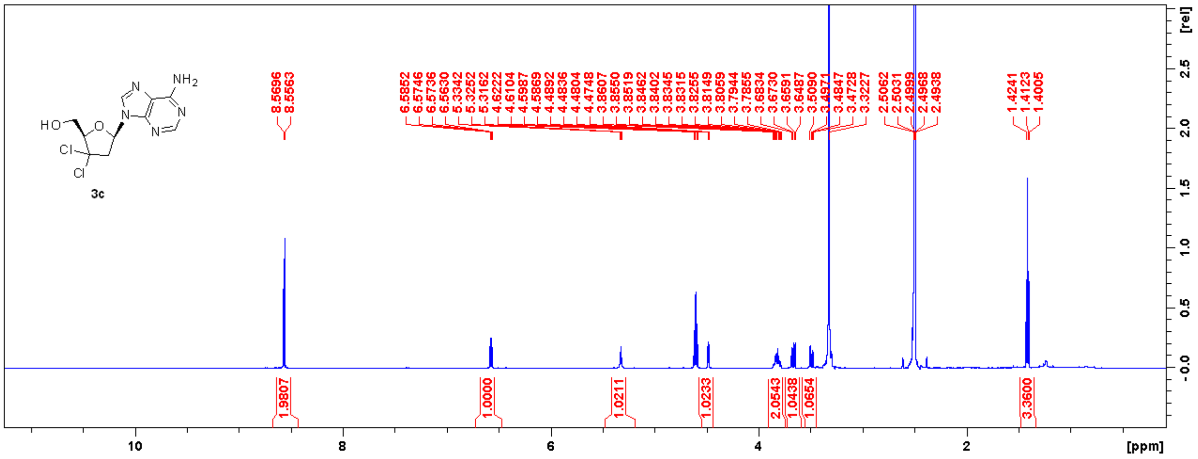

^{13}C NMR (150 MHz, DMSO-*d6*) spectrum of Compound **23**.

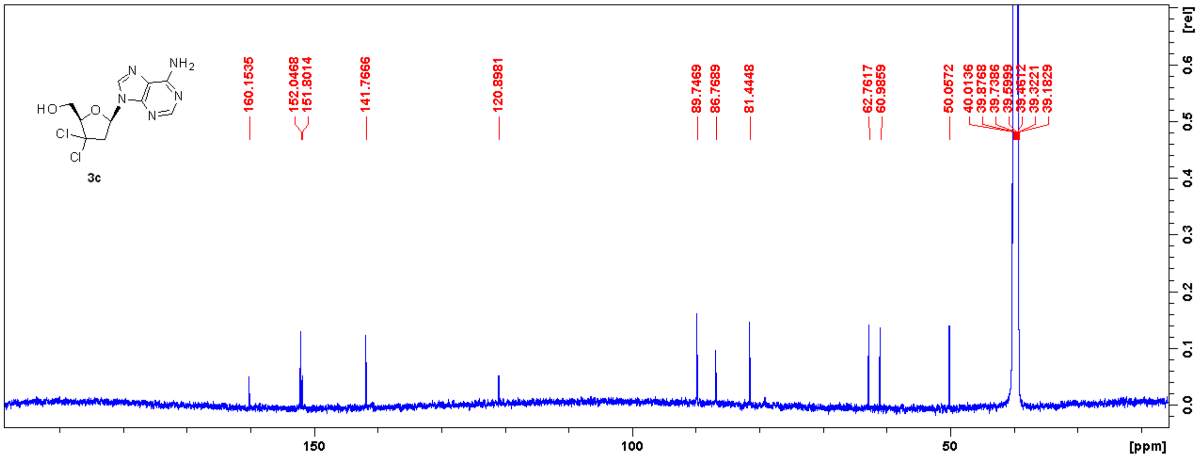

^{1}H NMR (300 MHz, MeOD-*d4*) spectrum of Compound **26.**

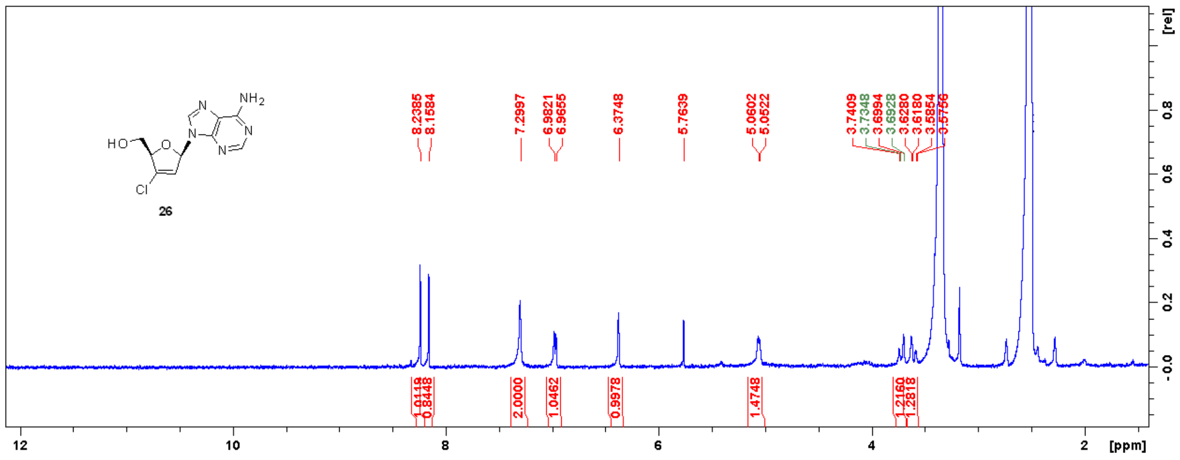

^{1}H NMR (75 MHz, MeOD-*d4*) spectrum of Compound **26.**

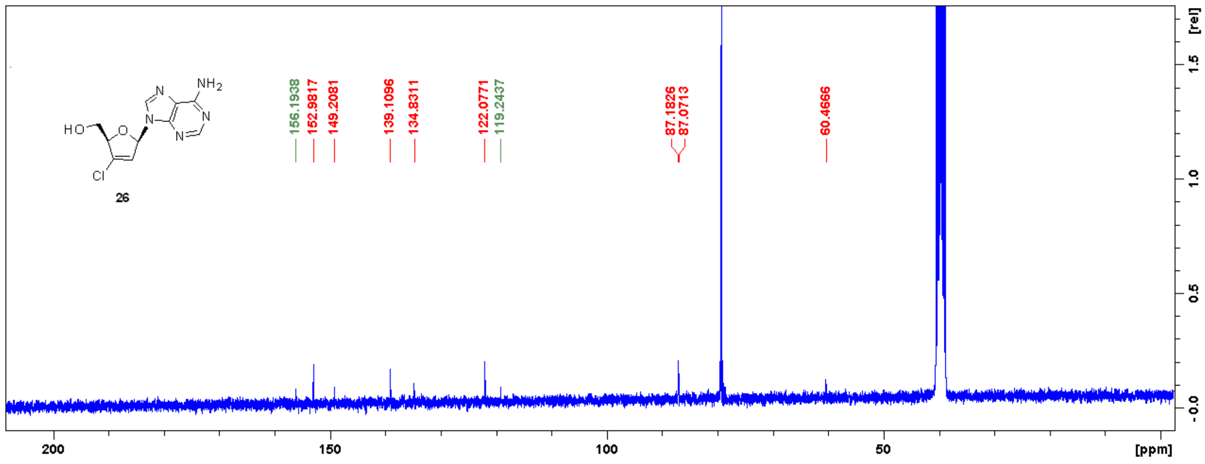

^{1}H NMR (600 MHz, MeOD-*d4*) spectrum of Compound **4a**.

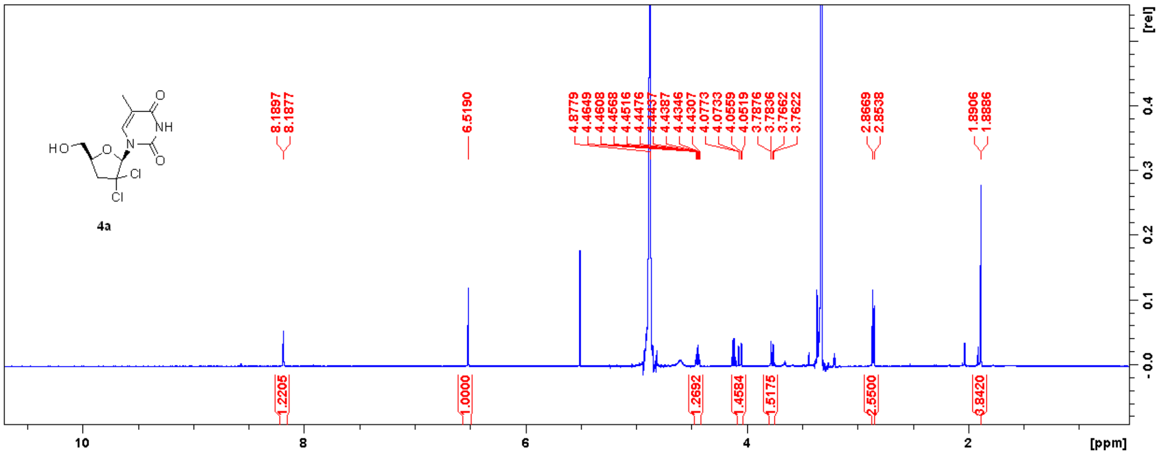

^{1}H NMR (150 MHz, MeOD-*d4*) spectrum of Compound **4a**.

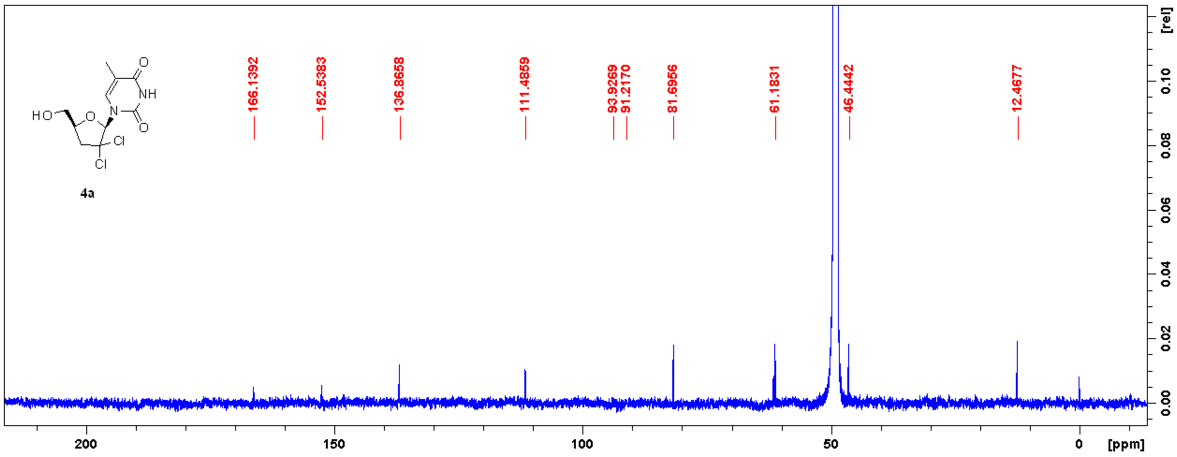

^{1}H NMR (600 MHz, MeOD-*d4*) spectrum of Compound **4b**.

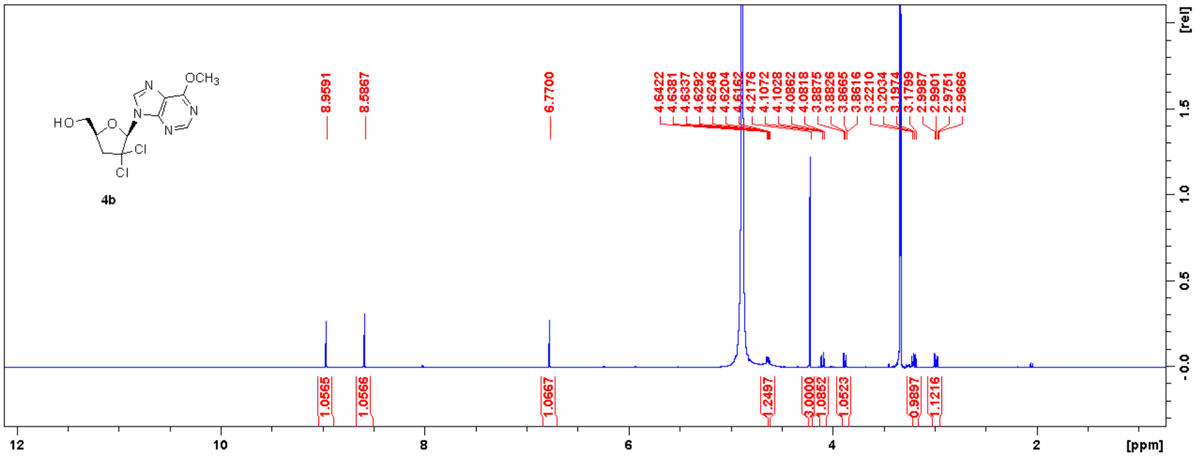

^{1}H NMR (150 MHz, MeOD-*d4*) spectrum of Compound **4b**.

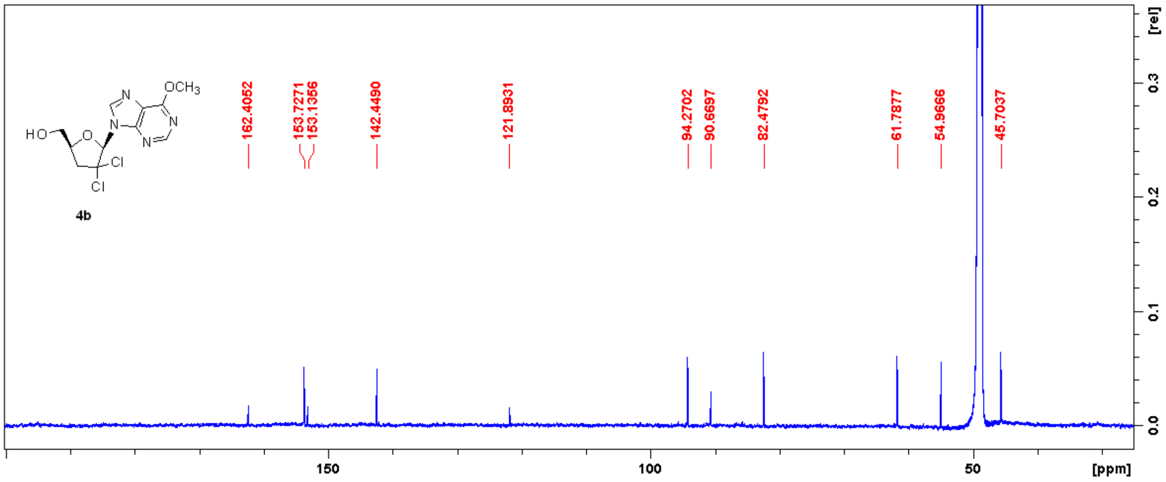

^{1}H NMR (600 MHz, MeOD-*d4*) spectrum of Compound **4c**.

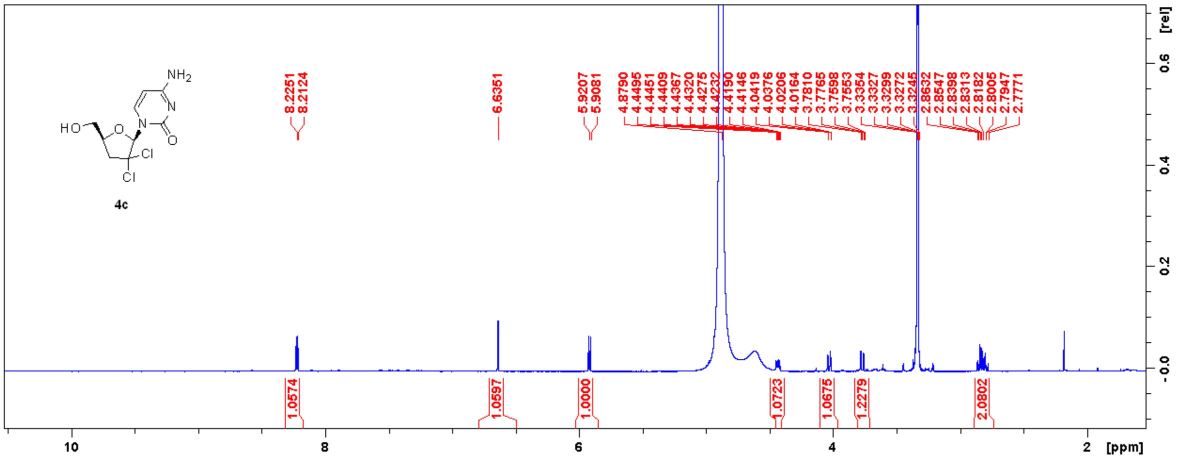

^{1}H NMR (150 MHz, MeOD-*d4*) spectrum of Compound **4c**.

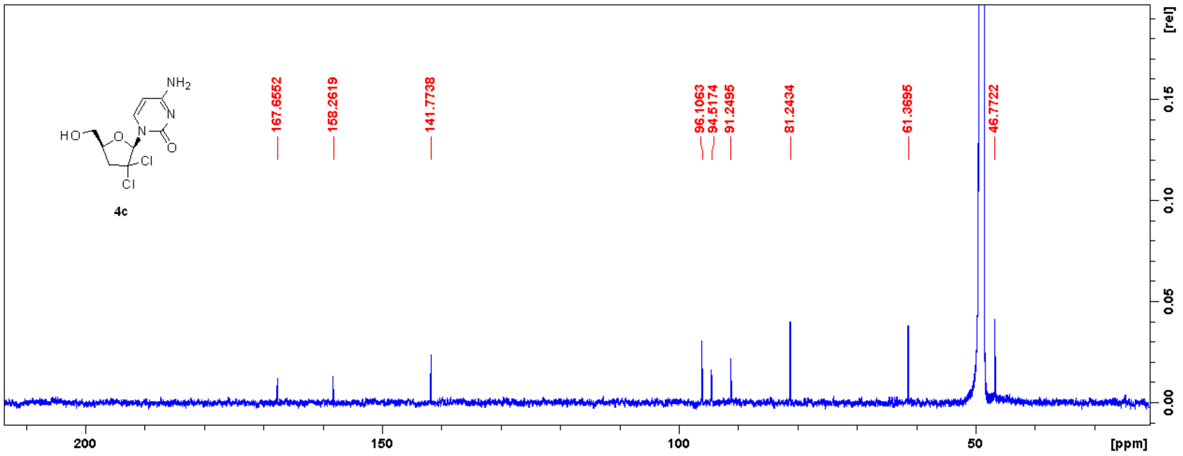

^{1}H NMR (300 MHz, MeOD-*d4*) spectrum of Compound **4d**.

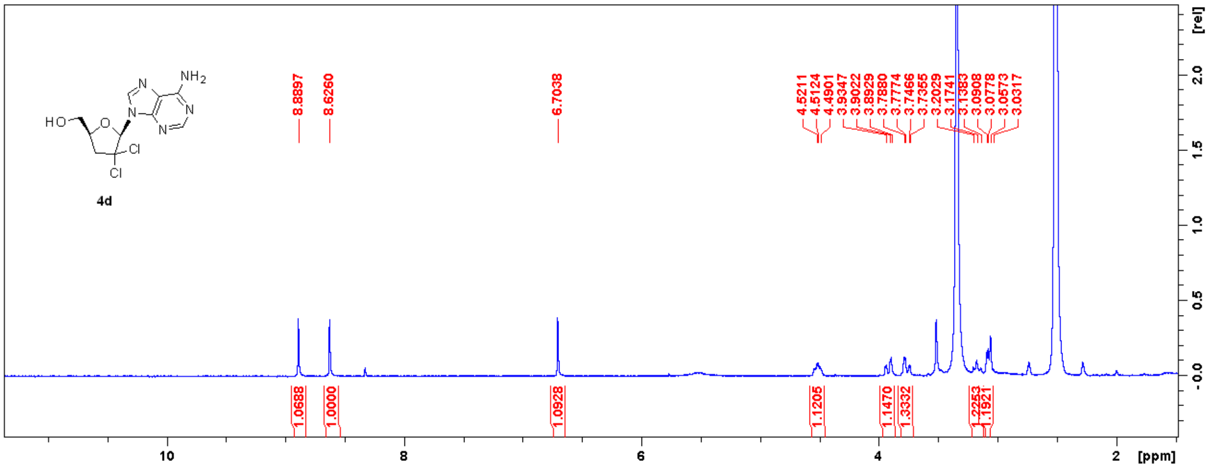

^{1}H NMR (75 MHz, MeOD-*d4*) spectrum of Compound **4d**.

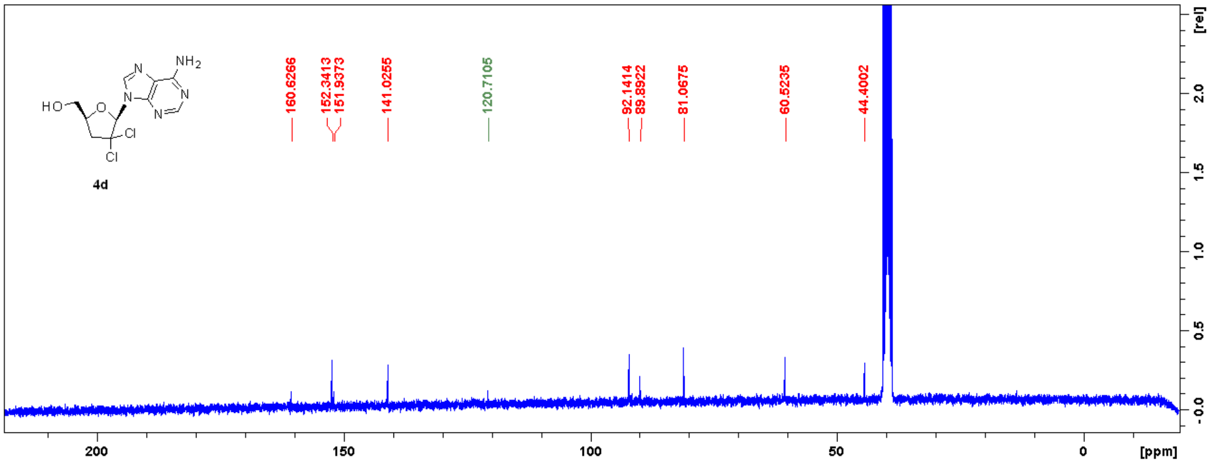

Supplement: Supplementary file 1 [file molecules-23-01457-s001.pdf]
